# *APOE* alleles modulate associations of plasma metabolites with variants from multiple genes on chromosome 19q13.3

**DOI:** 10.3389/fnagi.2022.1023493

**Published:** 2022-10-28

**Authors:** Alireza Nazarian, Elena Loiko, Hussein N. Yassine, Caleb E. Finch, Alexander M. Kulminski

**Affiliations:** ^1^Biodemography of Aging Research Unit, Social Science Research Institute, Duke University, Durham, NC, United States; ^2^Departments of Medicine and Neurology, Keck School of Medicine, University of Southern California, Los Angeles, CA, United States; ^3^Andrus Gerontology Center, University of Southern California, Los Angeles, CA, United States

**Keywords:** metabolomics, genetic heterogeneity, *APOE* ε2 allele, *APOE* ε3 allele, *APOE* ε4 allele, pleiotropy

## Abstract

The *APOE* ε2, ε3, and ε4 alleles differentially impact various complex diseases and traits. We examined whether these alleles modulated associations of 94 single-nucleotide polymorphisms (SNPs) harbored by 26 genes in 19q13.3 region with 217 plasma metabolites using Framingham Heart Study data. The analyses were performed in the E2 (ε2ε2 or ε2ε3 genotype), E3 (ε3ε3 genotype), and E4 (ε3ε4 or ε4ε4 genotype) groups separately. We identified 31, 17, and 22 polymorphism-metabolite associations in the E2, E3, and E4 groups, respectively, at a false discovery rate *P*_*FDR*_ < 0.05. These entailed 51 and 19 associations with 20 lipid and 12 polar analytes. Contrasting the effect sizes between the analyzed groups showed 20 associations with group-specific effects at Bonferroni-adjusted *P* < 7.14E−04. Three associations with glutamic acid or dimethylglycine had significantly larger effects in the E2 than E3 group and 12 associations with triacylglycerol 56:5, lysophosphatidylethanolamines 16:0, 18:0, 20:4, or phosphatidylcholine 38:6 had significantly larger effects in the E2 than E4 group. Two associations with isocitrate or propionate and three associations with phosphatidylcholines 32:0, 32:1, or 34:0 had significantly larger effects in the E4 than E3 group. Nine of 70 SNP-metabolite associations identified in either E2, E3, or E4 groups attained *P*_*FDR*_ < 0.05 in the pooled sample of these groups. However, none of them were among the 20 group-specific associations. Consistent with the evolutionary history of the *APOE* alleles, plasma metabolites showed higher *APOE*-cluster-related variations in the E4 than E2 and E3 groups. Pathway enrichment mainly highlighted lipids and amino acids metabolism and citrate cycle, which can be differentially impacted by the *APOE* alleles. These novel findings expand insights into the genetic heterogeneity of plasma metabolites and highlight the importance of the *APOE*-allele-stratified genetic analyses of the *APOE*-related diseases and traits.

## Introduction

The chromosome 19q13.3 harboring genes, such as *APOE* (apolipoprotein E), *NECTIN2* (nectin cell adhesion molecule 2), *TOMM40* (translocase of outer mitochondrial membrane 40), and *APOC1* (apolipoprotein C1) is involved in important physiological and pathological processes. The polymorphisms in this region have been implicated in Alzheimer’s disease (AD), cardiovascular and cerebrovascular pathologies, serum lipids, blood cells count, blood proteins, diabetes, obesity, longevity, etc. (MacArthur et al., 2017). In particular, the ε2, ε3, and ε4 alleles of *APOE* gene differentially impact the risk of various complex diseases and traits. AD, coronary artery disease (CAD), and lifespan are the most prominent examples, on which the ε4 allele has adverse effects whereas the ε2 allele confers beneficial effects compared with the ε3 allele (Belloy et al., 2019; Wolters et al., 2019). *APOE* encodes a lipoprotein, which plays key roles in lipid metabolism peripherally and in the central nervous system (CNS) (Stelzer et al., 2016). *APOE* alleles were found to differentially impact lipid metabolism (Wolters et al., 2019). The ε4 allele is considered as an atherogenic allele, which may be associated with increased risk of CAD and ischemic stroke *via* elevated serum levels of total cholesterol (TC) and low-density lipoprotein cholesterol (LDL-C), while ε2-carriers have lower plasma levels of TC and LDL-C and decreased risks of CAD and carotid artery atherosclerosis (Lehtimäki et al., 1990; Ilveskoski et al., 2000; Natarajan et al., 2016; Belloy et al., 2019; Karjalainen et al., 2019; Loika et al., 2021). In CNS, *APOE* is mainly involved in cholesterol homeostasis required for myelinization, neuronal membranes integrity and survival, and synaptogenesis. The ε4-encoded protein is maladaptive leading to changes in lipid metabolism and β-amyloid (Aβ) aggregation in brain due to its increased production and decreased clearance (Barberger-Gateau et al., 2011; Liu et al., 2013; Yamazaki et al., 2019).

In recent years, metabolomics analysis has emerged as a powerful strategy to identify molecular signatures of complex diseases and traits. The identified disease-metabolite associations can serve as novel disease biomarkers, which are important particularly for early diagnosis of late-onset diseases (Bäckström et al., 2003; Sabatine et al., 2005; Lewis et al., 2008; Gueli and Taibi, 2013; Laaksonen, 2016; Yanai, 2017; McGarrah et al., 2018; Socha et al., 2020; Piubelli et al., 2021). In addition, metabolites, as intermediate phenotypes, may mediate the genetic bases of complex disorders (Andreou et al., 2015). Interestingly, the differential impacts of *APOE* alleles have been reported in metabolomics studies as well. For instance, the healthy ε4 carriers were found to have significantly higher myoinositol-to-creatine and choline-to-creatine ratios compared to ε3ε3 subjects (Gomar et al., 2014). In a study of patients with early stages AD, eight metabolites, mostly glycerophospholipids (e.g., cardiolipins, lysophosphatidylcholines, and lysophosphatidylethanolamines) involved in lipid metabolism, could reliably discriminate ε4-carriers from non-carriers. All eight metabolites had lower serum levels in the ε4-carriers (Peña-Bautista et al., 2020). The differential impacts of ε2 and ε4 alleles on serum lipid metabolites have been also emphasized elsewhere (Karjalainen et al., 2019). As another example, while cortical Aβ aggregation was nominally associated with arachidonic acid, an ω-6 polyunsaturated fatty acid (PUFA), in ε4 non-carriers (*P* < 0.03), the similar association was non-significant in the ε4 carriers (*P* < 0.57) (Hooper et al., 2017). Randomized controlled trials have suggested that ε4 allele may alter the fate of ω-3 both in the brain (Arellanes et al., 2020) and in the periphery (Tomaszewski et al., 2020). For Instance, by examining the brain responses to high-dose supplementation (2.1 g/day over a 6-month period) with docosahexaenoic acid, Arellanes et al. (2020) found that the ε4 negative subjects had higher cerebrospinal fluid (CSF) levels of ω-3 docosahexaenoic acid and eicosapentaenoic acid compared to the ε4 positive subjects.

The *APOE* 19q13.3 locus is a genetically heterogenous region within which complex haplotype structures, interactions, and compound genotypes have been identified (Templeton et al., 2005; Yu et al., 2007; Lescai et al., 2011; Lutz et al., 2016; Babenko et al., 2018; Kulminski et al., 2018, 2020a,b,^2021^; Zhou et al., 2019; Nazarian et al., 2022a,b). For instance, linkage disequilibrium (LD) and association studies have highlighted complex genetic structure in this locus that are statistically different between AD-affected and unaffected subjects (Kulminski et al., 2018, 2020a,b). In addition, stratified analyses have identified sets of interactions and compound genotypes in the *APOE* locus of the ε2- and ε4-carriers, which may modify the effects of these alleles on AD risk and, to some extent, justify their incomplete penetrance (Nazarian et al., 2022a,b).

While previous studies have provided valuable insights into the genetic associations of the plasma metabolites (Mittelstrass et al., 2011; Rhee et al., 2013; Shin et al., 2014; Demirkan et al., 2015; Draisma et al., 2015; Krumsiek et al., 2015; Long et al., 2017; Lotta et al., 2021), little attention has been devoted to their genetic heterogeneity in the *APOE* 19q13.3 locus. Given the genetic complexity of this locus, we hypothesized that the *APOE* alleles may modulate associations of the regional single-nucleotide polymorphisms (SNPs) with plasma metabolites. Therefore, stratified analyses of SNP-metabolite associations in distinct groups of subjects carrying different *APOE* alleles/genotypes may reveal group-specific associations, which have not been identified in the non-stratified analyses. We examined associations between SNPs within the *APOE* locus and 217 plasma metabolites using data from the Framingham Heart Study (FHS) in three groups of subjects (i.e., E2: ε2ε2 and ε2ε3; E3: ε3ε3; and E4: ε3ε4 and ε4ε4). Our analyses revealed novel associations with plasma metabolites, mostly with lipid analytes. Two subsets of associations identified in ε2- or ε4-carriers were group-specific with significantly different effects between the groups under consideration.

## Materials and methods

### Study participants

We used the metabolite profiling data collected in the FHS Offspring cohort (Feinleib et al., 1975) and the genetic data from the FHS Candidate Gene Association Resource (CARe). The metabolite profiling data provides information on blood concentrations of 217 polar [e.g., amino acids, biogenic amines, carnitine, carbohydrates (e.g., pentose, hexoses, and disaccharides), nucleosides, etc.] and lipid [e.g., diacylglycerols (DAGs), triacylglycerols (TAGs), cholesterol esters (CEs), phosphatidylcholines (PCs), lysophosphatidylcholine (LPC), lysophosphatidylethanolamines (LPEs), and sphingomyelins (SMs)] analytes measured by a liquid chromatography/mass spectrometry (LC/MS) platform. The measured metabolites have been reported as normalized unit-free values (Feinleib et al., 1975; Rhee et al., 2013). To analyze the genetic heterogeneity at the *APOE* 19q13.3 locus, study participants were divided into three groups based on their *APOE* genotypes: (1) E2: subjects with ε2ε2 or ε2ε3 genotype, (2) E3: subjects with ε3ε3 genotype, and (3) E4: subjects with ε3ε4 or ε4ε4 genotype. The FHS study designated *APOE* genotypes of participants and their cognitive status based on the neurologic exam criteria (McKhann et al., 1984, 2011). Table 1 summarizes the basic demographic and clinical information about these 1798 subjects. The study participants were of Caucasian ancestry.

**TABLE 1:** Demographic information about study participants.

Group	*N*	F%	SMOKE%	AD%	Birth year	FBG	TC	LDL-C	HDL-C	TG	BMI	DBP	SBP	eGFR
ALL	1,798	53.00	61.40	4.34	1,935.12 (9.66)	95.97 (20.07)	203.81 (32.63)	128.07 (31.61)	50.42 (12.95)	119.57 (77.04)	26.28 (4.38)	77.36 (7.97)	122.79 (13.74)	66.46 (21.54)
E2	234	53.42	61.97	1.28	1,935.09 (9.79)	95.27 (15.86)	192.16 (32.16)	112.44 (30.75)	52.51 (14.48)	121.16 (68.43)	26.29 (4.35)	77.32 (8.19)	122.78 (14.35)	66.40 (16.26)
E3	1,185	52.83	62.19	3.54	1,935.14 (9.73)	95.98 (20.91)	203.93 (32.44)	129.16 (30.56)	50.39 (12.70)	116.07 (79.58)	26.29 (4.39)	77.25 (8.00)	122.70 (13.77)	66.06 (23.31)
E4	379	53.30	58.58	8.71	1,935.09 (9.37)	96.35 (19.73)	210.59 (31.58)	134.33 (32.35)	49.22 (12.57)	129.52 (73.14)	26.25 (4.37)	77.74 (7.72)	123.05 (13.26)	67.71 (18.42)

E2, ε2ε2 and ε2ε3 subjects; E3, ε3ε3 subjects; E4, ε3ε4 and ε4ε4 subjects; *N*, number of subjects; F%, percentage of females; SMOKE%, percentage of smokers; AD%, percentage of Alzheimer’s disease cases; FBG, fasting blood glucose (mg/dl); TC, total cholesterol (mg/dl); LDL-C, low-density lipoprotein cholesterol (mg/dl); HDL-C, high-density lipoprotein cholesterol (mg/dl); TG, triglycerides (mg/dl); BMI, body mass index (kg/m^2^); DBP, diastolic blood pressure (mmHg); SBP, systolic blood pressure (mmHg); eGFR, estimated glomerular filtration rate (ml/min/1.73 m^2^). The provided values for birth year, FBG, TC, LDL-C, HDL-C, TG, BMI, DBP, SBP, and eGFR represent their averages (and standard deviations).

### Genotype data quality control

The low-quality genetic data including SNPs with minor allele frequencies less than 1%; SNPs with *P*-values less than 1E−06 in Hardy-Weinberg equilibrium test; SNPs or subjects with missing rates larger than 5%; and SNPs, subjects, or families with Mendel error rates larger than 2% were first filtered out using *PLINK* package (Purcell et al., 2007). All SNPs in the *APOE* 19q13.3 locus (within 1 Mb up-/downstream of the *APOE* gene) available on the FHS CARe array were included. After quality control (QC) process, 94 SNPs (mapped to 26 genes from *CEACAM22P* to *RSPH6A*) were retained for our analyses.

### Association analysis

#### Stage one: Association study

Additive genetic models were fitted to identify the associations between the log-transformed concentrations of each of the 217 metabolites and the 94 SNPs in the *APOE* 19q13.3 locus using *GCTA* package (Yang et al., 2011). Following (Rhee et al., 2013; Huang et al., 2020), the fitted linear regression models were adjusted for sex, birth year, smoking history, body mass index (BMI), diastolic blood pressure (DBP), systolic blood pressure (SBP), fasting blood glucose (FBG), TC, LDL-C, high-density lipoprotein cholesterol (HDL-C), triglycerides (TG), estimated glomerular filtration rate (eGFR), and AD status of subjects, as well as the top five principal components of genetic data [obtained by the *GENESIS* R package (Conomos et al., 2015)] as fixed-effects covariates. Since the FHS dataset has a family-based design, the family structure was included as a random-effects covariate. Significant associations were identified at a false discovery rate (FDR) adjusted *P*_*FDR*_ < 0.05 (Benjamini and Hochberg, 1995; Storey et al., 2022). Stage one analyses were separately performed in each of the three *APOE*-stratified groups of participants (i.e., E2, E3, and E4 sub-groups).

#### Stage two: Group-specific associations

For any SNP significantly associated with a given metabolite in each of the E2, E3, and E4 groups, the effects (i.e., beta coefficients) of the SNP were contrasted between E2 and E3 groups, E4 and E3 groups, and E2 and E4 groups using a Chi-square test (Allison, 1999):


$$\chi^{2}=\frac{{\left(b_{1}-b_{2}\right)}^{2}}{{\text{SE}}_{1}^{2}+{\text{SE}}_{2}^{2}}$$

where, *b*_1_ (SE_1_) and *b*_2_ (SE_2_) are the beta coefficients (their standard errors) for a given SNP in the two contrasted groups.

Any SNP with significantly different effects between the two contrasted groups was considered to have a group-specific association. Significant findings from the chi-square tests were determined at a Bonferroni-adjusted threshold considering the number of tested SNPs.

Since the E2, E3, and E4 groups had different sample sizes, which would impact the statistical power of corresponding association analyses, the group-specific associations were further evaluated by fitting interaction models (Gogarten et al., 2019) in which the *APOE* status, SNP, and SNP-by-*APOE* status were included as fixed-effects variables along with the covariates mentioned in stage one analyses. A significant interaction term for any SNP corroborated its group-specific association.

### Pathway enrichment

*MetaboAnalyst (v5.0)* webtool (Pang et al., 2021) was used to identify *KEGG* (Kyoto Encyclopedia of Genes and Genomes) pathways (Kanehisa and Goto, 2000) enriched by metabolites that had significant associations in stage one of genetic analyses.

## Results

### Association analysis

Despite the importance of controlling for confounding effects of the cardiovascular and metabolic syndrome-related risk factors on genetic associations of plasma metabolites (Rhee et al., 2013; Huang et al., 2020), the inclusion of several covariates in the fitted model may increase the risk of multicollinearity. That is, if the variables of interest (here 94 SNPs in the *APOE* 19q13.3 locus) have high collinearity with other covariates, their estimated parameters would be unstable due to a potential decrease in the effective sample sizes (and statistical powers) and inflated standard errors (Johnston et al., 2018; Tomaschek et al., 2018). By examining potential multicollinearity, we found that these SNPs were weakly correlated with the other covariates (Pearson squared correlation coefficient *r*^2^ ranged from 1.30E−40 to 5.36E−02) and had small variance inflation factors (VIFs) of 1.01 to 1.36. These results implied minor impacts from multicollinearity on the estimated parameters, which can be safely ignored (Johnston et al., 2018; Tomaschek et al., 2018).

Our analyses identified 70 SNP-metabolite associations (with 20 lipid metabolites and 12 polar analytes) at *P*_*FDR*_ < 0.05 in the E2, E3, and E4 groups (Supplementary Tables 1–3). Most (51) of the identified associations were with lipid analytes, such as TAGs, LPEs, PCs, CEs, and SMs, many of which contained PUFAs [i.e., fatty acids (FAs) with two or more double bonds]. Several associations were also with non-lipid soluble analytes (e.g., amino acids, disaccharide, nucleosides, etc.).

Analyzing the E2 group, we found 31 SNP-metabolite associations (Supplementary Table 1). 27 of them were with lipid analytes including associations of SNPs mapped to *BCL3* and *APOE* with multiple TAGs (i.e., 54:4, 54:5, 54:6, 56:3, 56:4, and 56:5); mapped to *APOC2*, *APOC4*, and *CLPTM1* with three LPEs (i.e., 16:0, 18:0, 20:4); and mapped to *CKM* with PC 38:6. In addition, 4 SNP-metabolite associations were identified for non-lipid analytes including associations of rs10402271 (*BCAM* variant) with disaccharide sucrose (containing one glucose and one fructose molecules), rs10413089 (*APOC2* variant) and rs10421404 (*CLPTM1* variant) with amino acid glutamic acid, and rs16979759 (*CKM* variant) with dimethylglycine, a derivative of the amino acid glycine.

A total of 17 SNP-metabolite associations were identified in the E3 group (Supplementary Table 2), most (14) of which were with lipid analytes including associations of SNPs mapped to *APOC2*, *APOC4*, and *CLPTM1* with CE 20:3, PC 32:0, and mapped to *APOC4*/*APOC4-APOC2* with TAG 56:4. In addition, rs4884 (*CKM* variant) and rs11083777 (*EML2* variant) were associated with amino acids aspartate and asparagine, respectively, and rs16939 (*DMPK* variant) was associated with xanthosine, a purine nucleoside.

Our analyses identified 22 SNP-metabolite associations in the E4 group (Supplementary Table 3). 10 of them were with lipid analytes including associations of SNPs mapped to *NECTIN2* with three PCs (i.e., 32:0, 32:1, and 34:0); mapped to *NECTIN2* and *CKM* with two SMs (i.e., 18:0 and 22:0, respectively); and mapped to *NECTIN2*, *CKM*, *DMPK*, and *DMWD* with various TAGs (i.e., 50:2, 52:1, 56:6, and 58:11). Several associations with amino acids derivatives were also identified including associations of five SNPs (i.e., rs1871046, rs12610605, rs8106922, rs440446, and rs769450) mapped to *NECTIN2*, *TOMM40*, and *APOE* with ADMA (asymmetric dimethylarginine), a derivative of the amino acid L-arginine; and the association of rs12709889 (*APOC2*/*APOC4-APOC2* variant) with the thyroid gland hormone thyroxin, a derivative of the amino acid tyrosine. Also, three SNPs mapped to *NECTIN2* (i.e., rs519113, rs2075642, and rs387976) were associated with two intermediates of the tricarboxylic acid (TCA) cycle, i.e., aconitate and isocitrate. Finally, rs12609547 (*RELB* variant) was associated with uridine, a pyrimidine nucleoside and rs1064725 (*APOC1* variant) was associated with propionate, a conjugate base of a short-chain FA.

Most of the lipid metabolites with significant associations in the E2, E3, and E4 groups contained ω-3 (e.g., eicosatetraenoic acid and docosahexaenoic acid) or ω-6 (e.g., arachidonic acid) PUFAs in their structures (Wishart et al., 2007). These were more abundant in the E2 group than in the other two groups [i.e., TAGs 54:4, 54:5, 54:6, 56:3, 56:4, 56:5, LPE 20:4, PC 38:6 (E2 group); TAG 56:4, CE 20:3 (E3 group); and TAGs 50:2, 56:6, 58:11 (E4 group)].

#### *APOE* SNPs

The ε2-encoding SNP, rs7412, was associated with TAGs 54:5, 54:6, 56:3, and 56:4 in the E2 group. In contrast, no associations were identified for the ε4-encoding SNP, rs429358, in the E4 group at *P*_*FDR*_ < 0.05.

#### SNPs overlaps between groups

Several SNP associations were significant in both E2 and E3 groups (i.e., rs5157, rs2288912, rs7257468, rs3760627, and rs2239375), although their associations were with different lipid analytes. These SNPs were associated with LPEs 16:0, 18:0, and 20:4 in the E2 group but with CE 20:3 and PC 32:0 in the E3 group. Also, rs12709889 was associated with PC 32:0 in the E3 group and with thyroxine in the E4 group.

#### Linkage disequilibrium

In each group there are metabolites associated with multiple SNPs. Such associations may not be merely attributed to the potential LD between SNPs. For instance, while rs7412, rs17728272, and rs2965174 are associated with TAGs 54:5 and 54:6 in the E2 group, rs7412 is not in noticeable LD with these two SNPs (*r*^2^ = 0.01, *P* < 0.1595 and *r*^2^ = 0.0365, *P* < 0.0072, respectively). As another example, rs1871046 and rs2075642, which are among significant SNPs in the E4 group, are in significant LD (*r*^2^ = 0.1281, *P* < 0.0001), however, the former is associated with ADMA and the latter is associated with isocitrate. Supplementary Tables 4–6 display the LD measures (*r*^2^ in lower-left triangle and D′ in upper-right triangle) among the identified SNPs in the three analyzed groups. The SNP-pairs with *r*^2^ > 0.1 are in significant LD at *P* < 0.0001 in the CEU population (i.e., Utah Residents with Northern and Western European Ancestry) (Machiela and Chanock, 2015).

#### Group-specific associations

The Chi-square test to compare the effect sizes (i.e., beta coefficients) of SNPs between the three analyzed groups revealed that among associations identified in the E2 group, three SNPs-amino acids associations [i.e., the associations of rs10421404 (*APOC2*/*APOC4-APOC2* variant) and rs10413089 (*CLPTM1* variant) with glutamic acid, and rs16979759 (*CKM* variant) with dimethylglycine] were significantly different between the E2 and E3 groups at the Bonferroni-adjusted significance level of 7.14E−04 (i.e., 0.05/70). The effect directions of these three SNPs were opposite in the two groups, indicating opposite patterns of associations between their minor/major alleles and metabolites concentrations in the E2 and E3 groups. In addition, the effects of these SNPs were nominally different between the E2 and E4 groups. Also, 12 associations with lipid analytes, comprising of 6 SNPs [i.e., the association of rs2965101 and rs17728272 (*BCL3* variants) with TAG 56:5; rs7257468 (*APOC2*/*APOC4-APOC2* variant), rs3760627, and rs2239375 (*CLPTM1* variants) with LPEs 16:0, 18:0, and 20:4; and rs123187 (*CKM* variant) with PC 38:6] were significantly different between the E2 and E4 groups at *P* < 7.14E−04. Again, the effect directions of these SNPs were opposite in the two groups. Also, the effects of these SNPs were different between the E2 and E3 groups at nominal significance. The magnitudes of the estimated effects for these 15 SNPs were larger in the E2 group than the E3 or E4 group (Table 2 and Supplementary Table 1).

**TABLE 2:** Group-specific associations identified in the E2 group.

Phenotype	Gene	SNP	LOC	EA	E2			E3			E4			E2 vs. E3		E2 vs. E4	
					Beta	SE	*P*-value	Beta	SE	*P*-value	Beta	SE	*P*-value	χ^2^	*P*-value	χ^2^	*P*-value
TAG 56:5	*BCL3*	rs2965101	19:44734556	C	0.096	0.028	**7.27E−04**	−0.002	0.015	9.14E−01	−0.068	0.028	1.57E−02	9.274	2.32E−03	16.786	**4.18E−05**
TAG 56:5	*BCL3*	rs17728272	19:44737114	T	0.094	0.028	**7.15E−04**	−0.009	0.015	5.76E−01	−0.055	0.030	6.41E−02	10.455	1.22E−03	13.404	**2.51E−04**
Glutamic acid	*APOC2, APOC4-APOC2*	rs10421404	19:44949588	A	−0.271	0.079	**6.03E−04**	0.029	0.039	4.47E−01	0.026	0.064	6.88E−01	11.653	**6.41E−04**	8.472	3.61E−03
LPE 16:0	*APOC2, APOC4-APOC2*	rs7257468	19:44949887	T	−0.143	0.041	**4.17E−04**	−0.027	0.019	1.53E−01	0.046	0.029	1.17E−01	6.760	9.32E−03	14.270	**1.58E−04**
LPE 18:0	*APOC2, APOC4-APOC2*	rs7257468	19:44949887	T	−0.144	0.043	**8.17E−04**	0.000	0.017	9.90E−01	0.063	0.032	4.93E−02	9.615	1.93E−03	14.890	**1.14E−04**
LPE 20:4	*APOC2, APOC4-APOC2*	rs7257468	19:44949887	T	−0.131	0.036	**2.97E−04**	−0.022	0.015	1.46E−01	0.041	0.023	7.78E−02	7.648	5.68E−03	15.999	**6.34E−05**
Glutamic acid	*CLPTM1*	rs10413089	19:44952331	C	−0.271	0.079	**6.03E−04**	0.030	0.039	4.44E−01	0.026	0.064	6.88E−01	11.670	**6.35E−04**	8.472	3.61E−03
LPE 16:0	*CLPTM1*	rs3760627	19:44953923	C	−0.136	0.040	**7.30E−04**	−0.027	0.019	1.48E−01	0.047	0.029	1.10E−01	5.995	1.43E−02	13.469	**2.43E−04**
LPE 18:0	*CLPTM1*	rs3760627	19:44953923	C	−0.136	0.043	**1.45E−03**	−0.001	0.017	9.76E−01	0.063	0.032	4.78E−02	8.648	3.27E−03	13.954	**1.87E−04**
LPE 20:4	*CLPTM1*	rs3760627	19:44953923	C	−0.127	0.036	**4.05E−04**	−0.021	0.015	1.69E−01	0.040	0.023	8.21E−02	7.366	6.65E−03	15.344	**8.96E−05**
LPE 16:0	*CLPTM1*	rs2239375	19:44956594	C	−0.136	0.040	**7.30E−04**	−0.027	0.019	1.50E−01	0.040	0.029	1.71E−01	6.020	1.41E−02	12.508	**4.05E−04**
LPE 18:0	*CLPTM1*	rs2239375	19:44956594	C	−0.136	0.043	**1.45E−03**	−0.001	0.017	9.52E−01	0.062	0.032	5.34E−02	8.583	3.39E−03	13.746	**2.09E−04**
LPE 20:4	*CLPTM1*	rs2239375	19:44956594	C	−0.127	0.036	**4.05E−04**	−0.020	0.015	1.82E−01	0.034	0.023	1.43E−01	7.459	6.31E−03	14.215	**1.63E−04**
PC 38:6	*CKM*	rs123187	19:45327689	A	0.101	0.029	**5.23E−04**	0.000	0.011	9.91E−01	−0.029	0.021	1.67E−01	10.509	1.19E−03	13.090	**2.97E−04**
Dimethylglycine	*CKM*	rs16979759	19:45327876	G	0.813	0.196	**3.36E−05**	−0.102	0.058	8.06E−02	0.148	0.082	7.14E−02	20.013	**7.69E**−**06**	9.778	1.77E−03

E2, ε2ε2 and ε2ε3 subjects; E3, ε3ε3 subjects; E4, ε3ε4 and ε4ε4 subjects; SNP, single-nucleotide polymorphism; LOC, location of SNP in the chromosome:base-pair format based on Human Genome version 38 (hg38); EA, effect allele; EAF, effect allele frequency; Beta and SE, effect size and its standard error; χ^2^, Chi-square statistic corresponding to the comparison of effect sizes; TAG, triacylglycerol; LPE, lysophosphatidylethanolamine; PC, phosphatidylcholine. More details are given in Supplementary Table 1. Bold *p*-values indicate significant findings.

None of the SNP-metabolite associations identified in the E3 had group-specific effects in the chi-square test (Supplementary Table 2).

Among the SNP-metabolite associations identified in the E4 group, the associations of rs8105340 (*NECTIN2* variant) with PCs 32:0, 32:1, and 34:0; rs519113 (*NECTIN2* variant) with isocitrate; and rs1064725 (*APOC1* variant) with propionate were significantly different between the E4 and E3 groups at the Bonferroni-adjusted level of *P* < 7.14E−04. The directions of effects for the associations of rs8105340 with the aforementioned three PCs were opposite in the E3 and E4 groups, however, the two other SNPs had the same directions of effects in the two groups. In addition, the effects of SNPs in the latter two associations were different between the E2 and E4 groups at nominal significance. The magnitudes of the estimated effects for these five SNPs were larger in the E4 group than the E3 group (Table 3 and Supplementary Table 3).

**TABLE 3:** Group-specific associations identified in the E4 group.

Phenotype	Gene	SNP	LOC	EA	E2			E3			E4			E4 vs. E2		E4 vs. E3	
					Beta	SE	*P*-value	Beta	SE	*P*-value	Beta	SE	*P*-value	χ^2^	*P*-value	χ^2^	*P*-value
PC 32:1	*NECTIN2*	rs8105340	19:44864520	C	0.035	0.084	6.77E−01	−0.031	0.033	3.53E−01	0.201	0.054	**1.80E−04**	2.778	9.56E−02	13.499	**2.39E−04**
PC 32:0	*NECTIN2*	rs8105340	19:44864520	C	0.040	0.039	3.07E−01	−0.007	0.017	6.80E−01	0.112	0.028	**5.93E−05**	2.208	1.37E−01	13.316	**2.63E−04**
PC 34:1	*NECTIN2*	rs8105340	19:44864520	C	0.022	0.028	4.45E−01	−0.015	0.011	1.56E−01	0.073	0.018	**3.59E−05**	2.344	1.26E−01	18.237	**1.95E−05**
Isocitrate	*NECTIN2*	rs519113	19:44873027	G	0.026	0.029	3.77E−01	0.008	0.016	5.97E−01	0.111	0.025	**1.30E−05**	4.914	2.66E−02	11.833	**5.82E−04**
Propionate	*APOC1*	rs1064725	19:44919304	G	0.321	0.251	2.00E−01	−0.012	0.077	8.77E−01	−0.711	0.189	**1.70E−04**	10.803	1.01E−03	11.716	**6.20E−04**

E2, ε2ε2 and ε2ε3 subjects; E3, ε3ε3 subjects; E4, ε3ε4 and ε4ε4 subjects; SNP, single-nucleotide polymorphism; CHR, chromosome; LOC, location of SNP in the chromosome:base-pair format based on Human Genome version 38 (hg38); EA, effect allele; EAF, effect allele frequency; Beta and SE, effect size and its standard error; χ^2^, Chi-square statistic corresponding to the comparison of effect sizes; PC, phosphatidylcholine. More details are given in Supplementary Table 3. Bold *p*-values indicate significant findings.

The interaction analyses corroborated the group-specific associations as all 20 interaction terms (i.e., SNP-by-*APOE* status terms) were significant (Supplementary Table 7). Of note, the interaction of 18 SNPs with *APOE* status attained the Bonferroni-adjusted *P* < 7.14E−04. Two exceptions were the associations of rs123187 with PC 38:6 (significantly different between E2 and E4 groups) and rs519113 with isocitrate (significantly different between E3 and E4 groups) whose interactions with the *APOE* status attained *P* < 8.49E−04 and *P* < 1.13E−03, respectively.

None of the effects of the group-specific SNPs were significantly different between males and females in chi-square tests (*P* > 0.05), indicating minimal impacts of biological sex on the identified associations.

Supplementary Table 8 and Supplementary Figure 1 summarizes information on the statistical distributions of metabolites with group-specific associations. The means of these 12 metabolites were significantly different from zero indicating that the identified associations were not pointing to the technical measurement variability, which may particularly be important in the case of low-abundance metabolites. Also, their means were not different among the E2, E3, and E4 groups (except TAG 56:5), as indicated by overlapping confidence intervals for a given metabolite in these groups.

#### Association analysis in the pooled E2, E3, and E4 sample

Among 70 SNP-metabolite associations identified in the E2, E3, or E4 group, only 9 associations attained *P*_*FDR*_ < 0.05 in the pooled sample of these groups (Supplementary Table 9). They included associations of rs7412 with TAGs 56:3 and 56:4 (primarily identified in the E2 group), rs5157, rs5167, rs2288912, rs7257468, rs3760627, and rs2239375 with CE 20:3, and rs12721109 with TAG 56:4 (primarily identified in the E3 group). None of them were among the 20 group-specific associations.

The pooled sample analyses (Supplementary Table 10) showed that the *APOE* SNPs (rs429358 and rs7412) were not associated with 32 metabolites of interest at *P*_*FDR*_ < 0.05, except for the associations of rs7412 with TAGs 56:3 and 56:4.

#### *APOE*-allele-specific clustering of the identified associations

**FIGURE 1:**
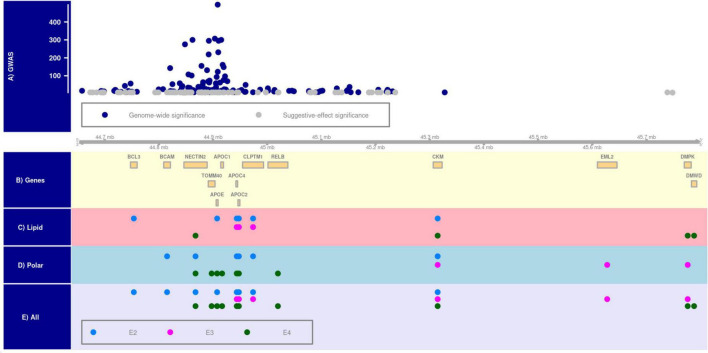
*APOE*-allele-specific clustering of genes identified for all metabolites, lipid metabolites, and polar metabolites. Panel **(A)** shows the distribution of –log (*p*-value) of Alzheimer’s disease-associated SNPs at genome-wide (i.e., *P* < 5E–08) and suggestive-effect (i.e., 5E–08 ≤ *P* < 5E–06) significance levels in the 19q13.3 region [chromosomal positions are based on Human Genome version 38 (hg38) and shown in mega base-pair format]. Panel **(B)** shows the location of genes in this locus. Panels **(C–E)** show metabolite-by-gene associations for polar, lipid, and all metabolites in the E2 (ε2ε2 and ε2ε3 subjects), E3 (ε3ε3 subjects), and E4 (ε3ε4 and ε4ε4 subjects) groups.

SNPs associated with plasma metabolites were mapped to 8, 6, and 10 genes in the E2, E3, and E4 groups, respectively. Of them, 2, 1, and 4 genes were uniquely linked to these groups, respectively Figure 1 [plotted using *Gviz* R package (Hahne and Ivanek, 2016)] and Supplementary Table 11. The clustering analysis of associations of lipid and polar metabolites separately showed that 6, 3, and 4 genes from the *APOE* region were associated with lipid metabolites in the E2, E3, and E4 groups, respectively. Of them, 2 and 3 genes were exclusively linked to the E2 and E4 groups, respectively. In addition, SNPs mapped to 6, 3, and 7 genes were associated with polar analytes in the E2, E3, and E4 groups, respectively, of which the associations of 2, 2, and 4 genes were unique in these groups, respectively.

### Pathway enrichment

The *KEGG* pathways enriched by lipid and polar metabolites, which were associated with *APOE* region SNPs in the E2, E3, or E4 groups, are displayed in Supplementary Figures 2–7. Most of the identified genetic associations (Supplementary Tables 1–3) were with lipid metabolites containing saturated and unsaturated FAs in their structures (Wishart et al., 2007) involved in lipid metabolism pathways, such as *biosynthesis of unsaturated fatty acids*, *fatty acid elongation*, and *fatty acid degradation pathways* (Supplementary Figures 2, 4, 6). Due to small number of polar metabolites with significant associations (3, 3, and 6 metabolites in the E2, E3, and E4 groups, respectively), most of pathways enriched by them (Supplementary Figures 3, 5, 7) contained only one metabolite. The top enriched pathways containing two or more polar analytes included *D-glutamine and D-glutamate metabolism* (E2 group); *alanine, aspartate and glutamate metabolism*, and *aminoacyl-tRNA biosynthesis* (E3 group); *tricarboxylic acid cycle (TCA, also known as citrate cycle)*, and *glyoxylate and dicarboxylate metabolism* (E4 group) (Table 4).

**TABLE 4:** Top pathways enriched by non-lipid metabolites in the E2, E3, and E4 groups.

Metabolite set	Total	Hits	*P*-value	*q*-value
**E2**				
D-Glutamine and D-glutamate metabolism	6	2	3.88E−05	3.26E−03
**E3**				
Alanine, aspartate, and glutamate metabolism	28	2	9.69E−04	8.14E−02
Aminoacyl-tRNA biosynthesis	48	2	2.86E−03	1.20E−01
**E4**				
Citrate cycle (TCA cycle)	20	2	2.39E−03	2.00E−01
Glyoxylate and dicarboxylate metabolism	32	2	6.10E−03	2.56E−01

E2, ε2ε2 and ε2ε3 subjects; E3, ε3ε3 subjects; E4, ε3ε4 and ε4ε4 subjects; TCA, tricarboxylic acid.

## Discussion

The *APOE* alleles differentially contribute to several complex diseases and traits. We performed stratified univariate analysis on the associations of 94 SNPs in the *APOE* gene cluster with 217 available plasma metabolites, to understand how *APOE* alleles may modify these associations. Our analyses revealed 31, 17, and 22 SNP-metabolite associations in the E2, E3, and E4 groups, respectively (Supplementary Tables 1–3). Of these 70 associations, 51 were with 20 lipid metabolites and 19 with 12 polar analytes. None of these associations were previously reported in samples not stratified by *APOE* alleles at the genome-wide (*P* < 5E−08) or suggestive-effect (5E−08 ≤ *P* < 5E−06) significance levels (Mittelstrass et al., 2011; Rhee et al., 2013; Shin et al., 2014; Demirkan et al., 2015; Draisma et al., 2015; Krumsiek et al., 2015; Long et al., 2017; Lotta et al., 2021).

We reviewed the GWAS catalog to identify links between metabolite-associated SNPs and other phenotypes (MacArthur et al., 2017). In addition to AD, we uncovered extensive associations with lipid traits. In the E2 group, rs10402271 was previously associated with TC, LDL-C, and HDL-C and rs10421404 was linked to LDL-C. Additionally, the ε2 encoding rs7412 showed extensive pleiotropy with TC, LDL-C, HDL-C, TG, apolipoprotein B (Apo-B), apolipoprotein A1 (Apo-A1), and lipoprotein (a). In the E3 group, we found rs12721109 was previously associated with TC, LDL-C, TG, Apo-B, Apo-A1, and lipoprotein (a). Furthermore, rs5167 showed associations with HDL-C and TG. In the E4 group, GWAS have identified associations of rs12610605 and rs1064725 with TG, rs519113 with HDL-C, rs8106922 with TC and LDL-C, and rs769450 with TC, LDL-C, Apo-B, and lipoprotein (a).

Interestingly, we found the effects of 15 SNP-metabolite associations for nine SNPs in the E2 group were significantly different between the E2 and E3 groups (3 associations) or the E2 and E4 groups (12 associations) (Table 2 and Supplementary Tables 1, 7). The former three associations were linked to two polar analytes (glutamic acid and dimethylglycine), whereas the latter 12 were linked to lipid analytes. Of these 12 associations, six were with LPE 20:4, PC 38:6, and TAG 56:5, which included ω-3 or ω-6 PUFAs in their structures (Wishart et al., 2007). In the E4 group, we found five SNP-metabolite associations for three SNPs with significantly different effects between the E4 and E3 groups (Table 3 and Supplementary Tables 3, 7). Three SNP-metabolite associations were with phosphatidylcholines, and the other two were with isocitrate and propionate polar analytes. None of these group-specific SNP-metabolite associations were significant in the pooled sample of the E2, E3, and E4 groups. The group-specific associations indicate genetic heterogeneity of plasma metabolites in the *APOE* 19q13.3 locus, which is in line with our previous studies that demonstrated complex LD structure differentially affects AD risk in this locus (Kulminski et al., 2018, 2020a,b; Nazarian et al., 2022a,b). Therefore, we suggest *APOE*-stratified analyses are essential for dissecting the genetic architecture of complex diseases and traits sensitive to *APOE* ε2/ε3/ε4 polymorphism.

Allele-specific clustering of the identified associations showed higher genetic variation of plasma metabolites in the E4 group compared to the E2 and E3 groups (Figure 1 and Supplementary Table 11), which may reflect evolutionary adaptation. Indeed, it is hypothesized that the ε3 allele evolved from the ancestral ε4 allele about 0.266 million years ago due to adaptation to meet consumption (Finch, 2012), followed by the ε2 allele (McIntosh et al., 2012; Huebbe and Rimbach, 2017). Consequently, more genetic variability and less robustness are expected in genetic networks linked to the ε4 allele compared to the other two alleles. Interestingly, the E4-associated genetic variations did not overlap with that of the E3 group in lipid and polar metabolites. This observation may suggest diverging molecular mechanisms in evolution of the ε3 allele.

Most of the identified genetic associations in our study were with lipid metabolites, many of which contained PUFAs (Wishart et al., 2007). Altered lipid metabolism has been implicated in the pathogenesis of diseases whose risks are modified by *APOE* ε2/ε3/ε4 polymorphism, such as AD (Zhu et al., 2019; Castor et al., 2020; Yin, 2022) and CAD (Freeman, 2006; Park et al., 2015; Cohain et al., 2021). Associations with PUFA-containing metabolites were more abundant in the E2 group than in the other two groups. PUFAs and their derivatives mediate FA signaling and are essential for neuron survival and function (Bazinet and Layé, 2014), and are involved in cellular immunity and neuroinflammation (Bazinet and Layé, 2014; Panda et al., 2022). Multiple studies have suggested ω-3 PUFA intake or supplementation can attenuate cognitive decline and dementia, particularly in people with sub-optimal plasma levels (Tully et al., 2003; Schaefer et al., 2006; Cherubini et al., 2007; Lopez et al., 2011; Yamagishi et al., 2017; Yanai, 2017; Hooper et al., 2018). Additionally, ω-3 PUFAs may be cardioprotective (Siscovick et al., 2017; Pizzini et al., 2018).

In addition, polar metabolites associated with *APOE* region SNPs in the E2, E3, and E4 groups have been implicated in AD (Jiménez-Jiménez et al., 1998; Selley, 2003; Dobolyi et al., 2011; Gueli and Taibi, 2013; Kaddurah-Daouk et al., 2013; Wang et al., 2014; Ansoleaga et al., 2015; González-Domínguez et al., 2015; Luo et al., 2015; Syeda et al., 2018; Baumel et al., 2021) and CAD (Valkonen et al., 2001; Bäckström et al., 2003; Lewis et al., 2008; Tveitevåg Svingen et al., 2013; Bartolomaeus et al., 2019; Cappola et al., 2019). For instance, AD patients had higher CSF levels (Jiménez-Jiménez et al., 1998) and a decreased cortical (temporal cortex) concentration (Gueli and Taibi, 2013) of glutamate. AD was also associated with lower plasma and CSF asparagine levels (Jiménez-Jiménez et al., 1998), as well as higher aspartate concentration in plasma (Jiménez-Jiménez et al., 1998) and lower aspartate concentration in the temporal cortex (Gueli and Taibi, 2013). Furthermore, AD was associated with increased plasma ADMA levels (Selley, 2003; Luo et al., 2015), as well as higher xanthosine in CSF (Kaddurah-Daouk et al., 2013) and lower xanthosine concentration in the entorhinal cortex (Ansoleaga et al., 2015). Also, AD mouse models had elevated propionate concentrations in the prefrontal cortex (Syeda et al., 2018) and hippocampus (González-Domínguez et al., 2015). In contrast, uridine dietary supplementation may slow brain atrophy and improve cognition in early AD stages (Dobolyi et al., 2011; Baumel et al., 2021).

Differential pathways enrichment of polar metabolite between *APOE* allele groups, such as *enrichment of D-glutamine and D-glutamate metabolism* in the E2 group and *TCA cycle* and *glyoxylate and dicarboxylate metabolism* in the E4 group (Table 4), is in line with previous reports that *APOE* alleles differentially impact the efficiency of various cellular metabolic processes (Konttinen et al., 2019; Williams et al., 2020). The ε4 allele is associated with decreased glucose uptake, and increased processes like lactate production from anaerobic glycolysis, glucose flux into the pentose phosphate pathway, gluconeogenesis (Williams et al., 2020), and utilization of PUFAs for energy production through calcium-dependent activation of phospholipase A2 signaling (Wang et al., 2022). Different brain regions of cognitively normal ε4 carriers have shown glucose hypometabolism, similar to AD patients (Reiman et al., 1996, 2004). In addition, the ε4 allele contributes to a higher pyruvate carboxylase to pyruvate dehydrogenase activity ratio and enrichment of glucose-derived carbon in TCA cycle. In contrast, the ε2 allele, and to some extent the ε3 allele, is associated with more efficient glucose uptake, increased glucose flux into glycolysis and oxidative TCA metabolism, decreased pyruvate carboxylase to pyruvate dehydrogenase activity ratio, and increased glutaminase enzyme levels which affect utilization of glutamine in TCA cycle (Williams et al., 2020). These metabolic differences are critical because disruption of mitochondrial glucose metabolism increases FAs metabolism, which can expose neurons to oxidative damage from PUFAs and monounsaturated FAs catabolism and increase the levels of oxidized dicarboxylic acids (DCAs) in urine. Indeed, elevated urinary excretion of DCAs may be a biomarker for early AD (Castor et al., 2020).

Despite the rigor, our study has limitations. The statistical power of our association analyses in the E2 and E4 groups was not optimal because the frequencies of the ε2 and ε4 alleles are substantially smaller in the general population than the frequency of the ε3 alleles. This disproportion may increase the number of false negatives in the E2 and E4 groups compared with the E3 group. However, this problem is partly offset by the significant findings in the comparative analysis of the group-specific effects. In addition, since we were interested in the potential modulatory effects of the *APOE* alleles on SNP-metabolite associations, the E2 and E4 groups included subjects with 1 or 2 copies of the ε2 and ε4 alleles, respectively. Future analyses may further refine these results by stratifying these two groups into heterozygotes and minor-allele homozygotes. Data with substantially larger sample sizes, however, is required for this analysis due to the small frequency of the ε2 and ε4 homozygotes in the general population. The analysis of independent studies will also help to replicate our findings and generalize them to other populations. Finally, the statistical power of our pathway enrichment analyses is not optimal due to the small number of metabolites having significant genetic associations.

## Conclusion

Our *APOE*-stratified analyses of the genetic heterogeneity of plasma metabolites identified 70 novel SNP-metabolite associations in the *APOE* 19q13.3 locus. Most of these associations were with lipid metabolites. The non-lipid metabolites were mainly enriched in pathways related to amino acid metabolism and the TCA cycle, which are differentially impacted by the *APOE* alleles. Consistent with the evolutionary history of the *APOE* alleles, the genetic architecture of plasma metabolites in the ε4 carriers entailed higher variation in the *APOE* genes cluster compared to the ε2 and ε3ε3 carriers. Importantly, twenty of these associations were found to be group-specific as the SNP effects were statistically different between subjects with different *APOE* alleles. These findings provide novel insights into the genetic heterogeneity of plasma metabolites at this locus and highlight the importance of the *APOE*-stratified analyses of diseases and traits differentially impacted by *APOE* alleles.

## Data availability statement

The FHS data used in this study can be obtained by qualified researchers from dbGaP (https://www.ncbi.nlm.nih.gov/gap/). The FHS data were accessed upon approval by the Duke University Institutional Review Board (IRB) and all analyses were performed following the IRB guidelines. Requests to access these datasets should be directed to https://www.ncbi.nlm.nih.gov/gap/.

## Ethics statement

This work focuses on the secondary analysis of data obtained from dbGaP upon approval by the Duke University Institutional Review Board (IRB) and does not involve gathering data from human subjects directly.

## Author contributions

AN and AMK designed the study. EL contributed to data preparation. AN analyzed the data. CEF and HNY provided the critical feedback. AN, AMK, CEF, and HNY wrote and revised the manuscript. All authors have read and approved the final manuscript.
