# Supplementary material for: APOE alleles modulate associations of plasma metabolites with variants from multiple genes on chromosome 19q13.3
Source: Front Aging Neurosci. 2022 Oct 28;14:1023493. doi: 10.3389/fnagi.2022.1023493 (PMC9650319; doi:10.3389/fnagi.2022.1023493)

**Supplementary Materials for:**

# *APOE* alleles modulate associations of plasma metabolites with variants from multiple genes on chromosome 19q13.3

Alireza Nazarian[1*], Elena Loiko[1], Hussein N. Yassine[2], Caleb E. Finch[3], and Alexander M. Kulminski[1*]

[1] Biodemography of Aging Research Unit, Social Science Research Institute, Duke University, Durham, NC, USA
[2] Departments of Medicine and Neurology, Keck School of Medicine, University of Southern California, Los Angeles, CA, USA
[3] Andrus Gerontology Center, University of Southern California, Los Angeles, CA, USA

*Corresponding Authors:
Alireza Nazarian and Alexander M. Kulminski
Duke University
Social Science Research Institute
Biodemography of Aging Research Unit
Erwin Mill Building, 2024 W. Main St.
Durham, NC 27705
Emails: alireza.nazarian@duke.edu and kulminsk@duke.edu

**This file includes:**

*Tables S1-S11* and *Figures S1-S7*

# Tables

**Table S1.** Associations identified in the E2 group.

| Phenotype | Gene | SNP | POS | EA | E2 | | | | | E3 | | | | | E4 | | | | | E2 vs. E3 | | | E2 vs. E4 | | |
|---|---|---|---|---|---|---|---|---|---|---|---|---|---|---|---|---|---|---|---|---|---|---|---|---|---|
| | | | | | EAF | beta | se | p-value | q-value | EAF | beta | se | p-value | q-value | EAF | beta | se | p-value | q-value | Magn | $\chi^2$ | p-value | Magn | $\chi^2$ | p-value |
| TAG 56:5 | *BCL3* | rs2965101 | 44734556 | C | 0.526 | 0.096 | 0.028 | **7.27E-04** | **3.34E-02** | 0.314 | -0.002 | 0.015 | 9.14E-01 | 9.98E-01 | 0.239 | -0.068 | 0.028 | 1.57E-02 | 4.99E-01 | E2 | 9.274 | 2.32E-03 | E2 | 16.786 | **4.18E-05** |
| TAG 54:6 | *BCL3* | rs17728272 | 44737114 | T | 0.304 | 0.094 | 0.032 | **3.61E-03** | **3.73E-02** | 0.261 | 0.000 | 0.016 | 9.75E-01 | 9.78E-01 | 0.203 | -0.044 | 0.030 | 1.43E-01 | 9.81E-01 | E2 | 6.929 | 8.48E-03 | E2 | 9.803 | 1.74E-03 |
| TAG 54:5 | *BCL3* | rs17728272 | 44737114 | T | 0.304 | 0.094 | 0.032 | **3.61E-03** | **3.73E-02** | 0.261 | 0.000 | 0.016 | 9.75E-01 | 9.78E-01 | 0.203 | -0.044 | 0.030 | 1.43E-01 | 9.81E-01 | E2 | 6.929 | 8.48E-03 | E2 | 9.803 | 1.74E-03 |
| TAG 56:5 | *BCL3* | rs17728272 | 44737114 | T | 0.304 | 0.094 | 0.028 | **7.15E-04** | **3.34E-02** | 0.261 | -0.009 | 0.015 | 5.76E-01 | 9.98E-01 | 0.203 | -0.055 | 0.030 | 6.41E-02 | 5.00E-01 | E2 | 10.455 | 1.22E-03 | E2 | 13.404 | **2.51E-04** |
| TAG 54:6 | *BCL3* | rs2965174 | 44741758 | A | 0.526 | -0.094 | 0.033 | **3.93E-03** | **3.73E-02** | 0.456 | 0.007 | 0.014 | 5.96E-01 | 9.78E-01 | 0.425 | 0.022 | 0.026 | 4.06E-01 | 9.81E-01 | E2 | 8.190 | 4.21E-03 | E2 | 7.701 | 5.52E-03 |
| TAG 54:5 | *BCL3* | rs2965174 | 44741758 | A | 0.526 | -0.094 | 0.033 | **3.93E-03** | **3.73E-02** | 0.456 | 0.007 | 0.014 | 5.96E-01 | 9.78E-01 | 0.425 | 0.022 | 0.026 | 4.06E-01 | 9.81E-01 | E2 | 8.190 | 4.21E-03 | E2 | 7.701 | 5.52E-03 |
| TAG 54:4 | *BCL3* | rs2965174 | 44741758 | A | 0.526 | -0.092 | 0.027 | **5.55E-04** | **4.32E-02** | 0.456 | 0.002 | 0.011 | 8.38E-01 | 9.78E-01 | 0.425 | 0.014 | 0.020 | 4.98E-01 | 9.75E-01 | E2 | 10.593 | 1.14E-03 | E2 | 10.046 | 1.53E-03 |
| Sucrose | *BCAM,NECTIN2* | rs10402271 | 44825957 | G | 0.187 | -0.489 | 0.133 | **2.51E-04** | **2.31E-02** | 0.315 | -0.027 | 0.040 | 4.98E-01 | 8.61E-01 | 0.467 | -0.110 | 0.092 | 2.32E-01 | 7.37E-01 | E2 | 10.976 | 9.23E-04 | E2 | 5.460 | 1.95E-02 |
| TAG 54:6 | *APOE* | rs7412 | 44908822 | T | 0.519 | 0.382 | 0.121 | **1.63E-03** | **3.73E-02** | 0 | NA | NA | NA | NA | 0 | NA | NA | NA | NA | NA | NA | NA | NA | NA | NA |
| TAG 54:5 | *APOE* | rs7412 | 44908822 | T | 0.519 | 0.382 | 0.121 | **1.63E-03** | **3.73E-02** | 0 | NA | NA | NA | NA | 0 | NA | NA | NA | NA | NA | NA | NA | NA | NA | NA |
| TAG 56:4 | *APOE* | rs7412 | 44908822 | T | 0.519 | 0.425 | 0.111 | **1.27E-04** | **1.17E-02** | 0 | NA | NA | NA | NA | 0 | NA | NA | NA | NA | NA | NA | NA | NA | NA | NA |
| TAG 56:3 | *APOE* | rs7412 | 44908822 | T | 0.519 | 0.580 | 0.143 | **4.99E-05** | **3.99E-03** | 0 | NA | NA | NA | NA | 0 | NA | NA | NA | NA | NA | NA | NA | NA | NA | NA |
| LPE 16:0 | *APOC4, APOC4-APOC2* | rs5157 | 44943904 | T | 0.455 | 0.128 | 0.039 | **8.73E-04** | **9.65E-03** | 0.537 | 0.030 | 0.018 | 1.07E-01 | 3.56E-01 | 0.498 | -0.020 | 0.029 | 4.85E-01 | 4.32E-01 | E2 | 5.301 | 2.13E-02 | E2 | 9.467 | 2.09E-03 |
| LPE 18:0 | *APOC4, APOC4-APOC2* | rs5157 | 44943904 | T | 0.455 | 0.122 | 0.041 | **3.04E-03** | **2.79E-02** | 0.537 | 0.012 | 0.017 | 4.97E-01 | 9.40E-01 | 0.498 | -0.034 | 0.032 | 2.84E-01 | 7.12E-01 | E2 | 6.133 | 1.33E-02 | E2 | 8.998 | 2.70E-03 |
| LPE 20:4 | *APOC4, APOC4-APOC2* | rs5157 | 44943904 | T | 0.455 | 0.115 | 0.035 | **8.71E-04** | **1.14E-02** | 0.537 | 0.018 | 0.015 | 2.37E-01 | 5.65E-01 | 0.498 | -0.006 | 0.023 | 7.90E-01 | 9.79E-01 | E2 | 6.639 | 9.98E-03 | E2 | 8.561 | 3.43E-03 |
| LPE 16:0 | *APOC2,APOC4, APOC4-APOC2* | rs2288912 | 44945942 | G | 0.548 | -0.131 | 0.039 | **7.38E-04** | **9.65E-03** | 0.456 | -0.033 | 0.019 | 7.51E-02 | 3.56E-01 | 0.498 | 0.023 | 0.029 | 4.34E-01 | 4.00E-01 | E2 | 5.203 | 2.25E-02 | E2 | 10.049 | 1.52E-03 |
| LPE 18:0 | *APOC2,APOC4, APOC4-APOC2* | rs2288912 | 44945942 | G | 0.548 | -0.125 | 0.041 | **2.51E-03** | **2.79E-02** | 0.456 | -0.011 | 0.017 | 5.13E-01 | 9.40E-01 | 0.498 | 0.039 | 0.032 | 2.15E-01 | 7.12E-01 | E2 | 6.475 | 1.09E-02 | E2 | 9.936 | 1.62E-03 |
| LPE 20:4 | *APOC2,APOC4, APOC4-APOC2* | rs2288912 | 44945942 | G | 0.548 | -0.122 | 0.035 | **4.45E-04** | **7.30E-03** | 0.456 | -0.025 | 0.015 | 1.01E-01 | 5.52E-01 | 0.498 | 0.014 | 0.023 | 5.48E-01 | 9.72E-01 | E2 | 6.603 | 1.02E-02 | E2 | 10.678 | 1.08E-03 |
| Glutamic acid | *APOC2, APOC4-APOC2* | rs10421404 | 44949588 | A | 0.210 | -0.271 | 0.079 | **6.03E-04** | **1.99E-02** | 0.165 | 0.029 | 0.039 | 4.47E-01 | 9.60E-01 | 0.194 | 0.026 | 0.064 | 6.88E-01 | 8.87E-01 | E2 | 11.653 | **6.41E-04** | E2 | 8.472 | 3.61E-03 |
| LPE 16:0 | *APOC2, APOC4-APOC2* | rs7257468 | 44949887 | T | 0.519 | -0.143 | 0.041 | **4.17E-04** | **9.65E-03** | 0.420 | -0.027 | 0.019 | 1.53E-01 | 3.56E-01 | 0.467 | 0.046 | 0.029 | 1.17E-01 | 2.51E-01 | E2 | 6.760 | 9.32E-03 | E2 | 14.270 | **1.58E-04** |
| LPE 18:0 | *APOC2, APOC4-APOC2* | rs7257468 | 44949887 | T | 0.519 | -0.144 | 0.043 | **8.17E-04** | **2.22E-02** | 0.420 | 0.000 | 0.017 | 9.90E-01 | 9.90E-01 | 0.467 | 0.063 | 0.032 | 4.93E-02 | 6.23E-01 | E2 | 9.615 | 1.93E-03 | E2 | 14.890 | **1.14E-04** |
| LPE 20:4 | *APOC2, APOC4-APOC2* | rs7257468 | 44949887 | T | 0.519 | -0.131 | 0.036 | **2.97E-04** | **7.30E-03** | 0.420 | -0.022 | 0.015 | 1.46E-01 | 5.52E-01 | 0.467 | 0.041 | 0.023 | 7.78E-02 | 9.72E-01 | E2 | 7.648 | 5.68E-03 | E2 | 15.999 | **6.34E-05** |
| Glutamic acid | *CLPTM1* | rs10413089 | 44952331 | C | 0.210 | -0.271 | 0.079 | **6.03E-04** | **1.99E-02** | 0.165 | 0.030 | 0.039 | 4.44E-01 | 9.60E-01 | 0.194 | 0.026 | 0.064 | 6.88E-01 | 8.87E-01 | E2 | 11.670 | **6.35E-04** | E2 | 8.472 | 3.61E-03 |
| LPE 16:0 | *CLPTM1* | rs3760627 | 44953923 | C | 0.516 | -0.136 | 0.040 | **7.30E-04** | **9.65E-03** | 0.420 | -0.027 | 0.019 | 1.48E-01 | 3.56E-01 | 0.469 | 0.047 | 0.029 | 1.10E-01 | 2.51E-01 | E2 | 5.995 | 1.43E-02 | E2 | 13.469 | **2.43E-04** |
| LPE 18:0 | *CLPTM1* | rs3760627 | 44953923 | C | 0.516 | -0.136 | 0.043 | **1.45E-03** | **2.22E-02** | 0.420 | -0.001 | 0.017 | 9.76E-01 | 9.90E-01 | 0.469 | 0.063 | 0.032 | 4.78E-02 | 6.23E-01 | E2 | 8.648 | 3.27E-03 | E2 | 13.954 | **1.87E-04** |
| LPE 20:4 | *CLPTM1* | rs3760627 | 44953923 | C | 0.516 | -0.127 | 0.036 | **4.05E-04** | **7.30E-03** | 0.420 | -0.021 | 0.015 | 1.69E-01 | 5.52E-01 | 0.469 | 0.040 | 0.023 | 8.21E-02 | 9.72E-01 | E2 | 7.366 | 6.65E-03 | E2 | 15.344 | **8.96E-05** |
| LPE 16:0 | *CLPTM1* | rs2239375 | 44956594 | C | 0.516 | -0.136 | 0.040 | **7.30E-04** | **9.65E-03** | 0.421 | -0.027 | 0.019 | 1.50E-01 | 3.56E-01 | 0.477 | 0.040 | 0.029 | 1.71E-01 | 2.51E-01 | E2 | 6.020 | 1.41E-02 | E2 | 12.508 | **4.05E-04** |
| LPE 18:0 | *CLPTM1* | rs2239375 | 44956594 | C | 0.516 | -0.136 | 0.043 | **1.45E-03** | **2.22E-02** | 0.421 | -0.001 | 0.017 | 9.52E-01 | 9.90E-01 | 0.477 | 0.062 | 0.032 | 5.34E-02 | 6.23E-01 | E2 | 8.583 | 3.39E-03 | E2 | 13.746 | **2.09E-04** |
| LPE 20:4 | *CLPTM1* | rs2239375 | 44956594 | C | 0.516 | -0.127 | 0.036 | **4.05E-04** | **7.30E-03** | 0.421 | -0.020 | 0.015 | 1.82E-01 | 5.52E-01 | 0.477 | 0.034 | 0.023 | 1.43E-01 | 9.72E-01 | E2 | 7.459 | 6.31E-03 | E2 | 14.215 | **1.63E-04** |
| PC 38:6 | *CKM* | rs123187 | 45327689 | A | 0.417 | 0.101 | 0.029 | **5.23E-04** | **4.81E-02** | 0.362 | 0.000 | 0.011 | 9.91E-01 | 9.91E-01 | 0.359 | -0.029 | 0.021 | 1.67E-01 | 6.42E-01 | E2 | 10.509 | 1.19E-03 | E2 | 13.090 | **2.97E-04** |
| Dimethylglycine | *CKM* | rs16979759 | 45327876 | G | 0.013 | 0.813 | 0.196 | **3.36E-05** | **3.13E-03** | 0.016 | -0.102 | 0.058 | 8.06E-02 | 8.69E-01 | 0.029 | 0.148 | 0.082 | 7.14E-02 | 9.89E-01 | E2 | 20.013 | **7.69E-06** | E2 | 9.778 | 1.77E-03 |

All SNPs are on chromosome 19q13.32.

Abbreviations: E2 = ε2ε2 and ε2ε3 subjects; E3 = ε3ε3 subjects; E4 = ε3ε4 and ε4ε4 subjects; SNP = single-nucleotide polymorphism; POS = SNP position based on Human Genome version 38 (hg38); EA = Effect allele; EAF = Effect allele frequency; beta and se = effect size and its standard error; Magn = the group in which the magnitude of SNP effect size (i.e., absolute value of beta) is larger; $\chi^2$ = chi-square statistic corresponding to the comparison of effect sizes; TAG = triacylglycerol; LPE = lysophosphatidylethanolamine; PC = phosphatidylcholine.

**Table S2.** Associations identified in the E3 group.

| Phenotype | Gene | SNP | POS | EA | E2 | | | | | E3 | | | | | E4 | | | | | E3 vs. E2 | | | E3 vs. E4 | | |
|---|---|---|---|---|---|---|---|---|---|---|---|---|---|---|---|---|---|---|---|---|---|---|---|---|---|
| | | | | | EAF | beta | se | p-value | q-value | EAF | beta | se | p-value | q-value | EAF | beta | se | p-value | q-value | Magn | $\chi^2$ | p-value | Magn | $\chi^2$ | p-value |
| PC 32:0 | *APOC4, APOC4-APOC2* | rs5157 | 44943904 | T | 0.455 | 0.032 | 0.022 | 1.49E-01 | 5.69E-01 | 0.537 | 0.031 | 0.010 | **1.53E-03** | **2.14E-02** | 0.498 | -0.008 | 0.018 | 6.70E-01 | 7.36E-01 | E2 | 0.002 | 9.67E-01 | E3 | 3.654 | 5.59E-02 |
| CE 20:3 | *APOC4, APOC4-APOC2* | rs5157 | 44943904 | T | 0.455 | -0.020 | 0.038 | 5.90E-01 | 7.99E-01 | 0.537 | -0.056 | 0.017 | **8.74E-04** | **1.77E-02** | 0.498 | -0.051 | 0.029 | 8.54E-02 | 9.68E-01 | E3 | 0.725 | 3.95E-01 | E3 | 0.025 | 8.76E-01 |
| TAG 56:4 | *APOC4, APOC4-APOC2* | rs12721109 | 44943964 | A | 0.106 | 0.113 | 0.054 | 3.48E-02 | 6.72E-01 | 0.009 | 0.282 | 0.075 | **1.70E-04** | **1.56E-02** | 0.008 | 0.079 | 0.142 | 5.79E-01 | 9.76E-01 | E3 | 3.363 | 6.67E-02 | E3 | 1.601 | 2.06E-01 |
| PC 32:0 | *APOC4, APOC4-APOC2* | rs5167 | 44945208 | G | 0.359 | -0.017 | 0.024 | 4.80E-01 | 5.97E-01 | 0.334 | -0.030 | 0.011 | **4.66E-03** | **4.65E-02** | 0.367 | -0.003 | 0.019 | 8.92E-01 | 7.66E-01 | E3 | 0.282 | 5.95E-01 | E3 | 1.652 | 1.99E-01 |
| CE 20:3 | *APOC4, APOC4-APOC2* | rs5167 | 44945208 | G | 0.359 | -0.031 | 0.040 | 4.44E-01 | 7.99E-01 | 0.334 | 0.057 | 0.018 | **1.41E-03** | **1.77E-02** | 0.367 | 0.086 | 0.031 | 5.66E-03 | 3.70E-01 | E3 | 4.035 | 4.46E-02 | E4 | 0.646 | 4.21E-01 |
| PC 32:0 | *APOC2,APOC4, APOC4-APOC2* | rs2288912 | 44945942 | G | 0.548 | -0.032 | 0.023 | 1.58E-01 | 5.69E-01 | 0.456 | -0.035 | 0.010 | **3.80E-04** | **1.12E-02** | 0.498 | 0.004 | 0.018 | 8.36E-01 | 7.66E-01 | E3 | 0.017 | 8.97E-01 | E3 | 3.641 | 5.64E-02 |
| CE 20:3 | *APOC2,APOC4, APOC4-APOC2* | rs2288912 | 44945942 | G | 0.548 | 0.020 | 0.038 | 5.94E-01 | 7.99E-01 | 0.456 | 0.055 | 0.017 | **1.14E-03** | **1.77E-02** | 0.498 | 0.049 | 0.029 | 9.60E-02 | 9.68E-01 | E3 | 0.668 | 4.14E-01 | E3 | 0.029 | 8.65E-01 |
| PC 32:0 | *APOC2, APOC4-APOC2* | rs7257468 | 44949887 | T | 0.519 | -0.036 | 0.024 | 1.23E-01 | 5.69E-01 | 0.420 | -0.036 | 0.010 | **3.24E-04** | **1.12E-02** | 0.467 | 0.011 | 0.018 | 5.36E-01 | 7.27E-01 | E3 | 0.000 | 9.99E-01 | E3 | 5.276 | 2.16E-02 |
| CE 20:3 | *APOC2 ,APOC4-APOC2* | rs7257468 | 44949887 | T | 0.519 | 0.023 | 0.040 | 5.68E-01 | 7.99E-01 | 0.420 | 0.053 | 0.017 | **1.75E-03** | **1.77E-02** | 0.467 | 0.035 | 0.030 | 2.43E-01 | 9.68E-01 | E3 | 0.499 | 4.80E-01 | E3 | 0.297 | 5.86E-01 |
| PC 32:0 | *APOC2, APOC4-APOC2* | rs12709889 | 44949982 | A | 0.283 | -0.013 | 0.026 | 6.21E-01 | 6.34E-01 | 0.240 | -0.036 | 0.013 | **4.26E-03** | **4.65E-02** | 0.228 | -0.019 | 0.024 | 4.25E-01 | 7.27E-01 | E3 | 0.611 | 4.34E-01 | E3 | 0.418 | 5.18E-01 |
| PC 32:0 | *CLPTM1* | rs3760627 | 44953923 | C | 0.516 | -0.032 | 0.023 | 1.66E-01 | 5.69E-01 | 0.420 | -0.035 | 0.010 | **5.96E-04** | **1.12E-02** | 0.469 | 0.010 | 0.018 | 5.71E-01 | 7.27E-01 | E3 | 0.008 | 9.27E-01 | E3 | 4.735 | 2.96E-02 |
| CE 20:3 | *CLPTM1* | rs3760627 | 44953923 | C | 0.516 | 0.019 | 0.039 | 6.28E-01 | 7.99E-01 | 0.420 | 0.054 | 0.017 | **1.66E-03** | **1.77E-02** | 0.469 | 0.033 | 0.030 | 2.65E-01 | 9.68E-01 | E3 | 0.641 | 4.23E-01 | E3 | 0.365 | 5.46E-01 |
| PC 32:0 | *CLPTM1* | rs2239375 | 44956594 | C | 0.516 | -0.032 | 0.023 | 1.66E-01 | 5.69E-01 | 0.421 | -0.034 | 0.010 | **6.44E-04** | **1.12E-02** | 0.477 | 0.005 | 0.018 | 7.99E-01 | 7.66E-01 | E3 | 0.007 | 9.35E-01 | E3 | 3.585 | 5.83E-02 |
| CE 20:3 | *CLPTM1* | rs2239375 | 44956594 | C | 0.516 | 0.019 | 0.039 | 6.28E-01 | 7.99E-01 | 0.421 | 0.054 | 0.017 | **1.63E-03** | **1.77E-02** | 0.477 | 0.032 | 0.030 | 2.78E-01 | 9.68E-01 | E3 | 0.641 | 4.23E-01 | E3 | 0.399 | 5.27E-01 |
| Aspartate | *CKM* | rs4884 | 45306777 | A | 0.295 | 0.064 | 0.079 | 4.16E-01 | 9.89E-01 | 0.285 | -0.114 | 0.031 | **2.25E-04** | **2.07E-02** | 0.302 | 0.034 | 0.062 | 5.84E-01 | 9.99E-01 | E3 | 4.439 | 3.51E-02 | E3 | 4.556 | 3.28E-02 |
| Asparagine | *EML2* | rs11083777 | 45654205 | A | 0.137 | 0.051 | 0.058 | 3.77E-01 | 7.09E-01 | 0.142 | -0.094 | 0.026 | **3.28E-04** | **2.18E-02** | 0.118 | -0.023 | 0.057 | 6.84E-01 | 9.24E-01 | E3 | 5.169 | 2.30E-02 | E3 | 1.278 | 2.58E-01 |
| Xanthosine | *DMPK* | rs16939 | 45772798 | A | 0.432 | 0.009 | 0.031 | 7.71E-01 | 9.63E-01 | 0.419 | -0.050 | 0.014 | **4.21E-04** | **3.70E-02** | 0.444 | 0.009 | 0.023 | 7.01E-01 | 9.99E-01 | E3 | 3.023 | 8.21E-02 | E3 | 4.694 | 3.03E-02 |

All SNPs are on chromosome 19q13.32.

Abbreviations: E2 = ε2ε2 and ε2ε3 subjects; E3 = ε3ε3 subjects; E4 = ε3ε4 and ε4ε4 subjects; SNP = single-nucleotide polymorphism; POS = SNP position based on Human Genome version 38 (hg38); EA = Effect allele; EAF = Effect allele frequency; beta and se = effect size and its standard error; Magn = the group in which the magnitude of SNP effect size (i.e., absolute value of beta) is larger; $\chi^2$ = chi-square statistic corresponding to the comparison of effect sizes; TAG = triacylglycerol; PC = phosphatidylcholine; CE = cholesterol ester.

**Table S3.** Associations identified in the E4 group.

| Phenotype | Gene | SNP | POS | EA | E2 | | | | | E3 | | | | | E4 | | | | | E4 vs. E2 | | | E4 vs. E3 | | |
|---|---|---|---|---|---|---|---|---|---|---|---|---|---|---|---|---|---|---|---|---|---|---|---|---|---|
| | | | | | EAF | beta | se | p-value | q-value | EAF | beta | se | p-value | q-value | EAF | beta | se | p-value | q-value | Magn | $\chi^2$ | p-value | Magn | $\chi^2$ | p-value |
| ADMA | *NECTIN2* | rs1871046 | 44848680 | C | 0.406 | -0.033 | 0.031 | 2.95E-01 | 7.95E-01 | 0.395 | 0.010 | 0.012 | 3.70E-01 | 7.09E-01 | 0.324 | 0.079 | 0.023 | **5.43E-04** | **2.09E-02** | E4 | 8.382 | 3.79E-03 | E4 | 7.186 | 7.35E-03 |
| PC 32:1 | *NECTIN2* | rs8105340 | 44864520 | C | 0.074 | 0.035 | 0.084 | 6.77E-01 | 9.93E-01 | 0.096 | -0.031 | 0.033 | 3.53E-01 | 6.48E-01 | 0.155 | 0.201 | 0.054 | **1.80E-04** | **1.20E-02** | E4 | 2.778 | 9.56E-02 | E4 | 13.499 | **2.39E-04** |
| PC 32:0 | *NECTIN2* | rs8105340 | 44864520 | C | 0.074 | 0.040 | 0.039 | 3.07E-01 | 5.69E-01 | 0.096 | -0.007 | 0.017 | 6.80E-01 | 7.20E-01 | 0.155 | 0.112 | 0.028 | **5.93E-05** | **4.43E-03** | E4 | 2.208 | 1.37E-01 | E4 | 13.316 | **2.63E-04** |
| PC 34:1 | *NECTIN2* | rs8105340 | 44864520 | C | 0.074 | 0.022 | 0.028 | 4.45E-01 | 3.05E-01 | 0.096 | -0.015 | 0.011 | 1.56E-01 | 7.61E-01 | 0.155 | 0.073 | 0.018 | **3.59E-05** | **2.88E-03** | E4 | 2.344 | 1.26E-01 | E4 | 18.237 | **1.95E-05** |
| SM 18:0 | *NECTIN2* | rs8105340 | 44864520 | C | 0.074 | 0.005 | 0.045 | 9.04E-01 | 9.90E-01 | 0.096 | 0.008 | 0.018 | 6.79E-01 | 5.00E-01 | 0.155 | 0.098 | 0.029 | **6.36E-04** | **4.46E-02** | E4 | 2.960 | 8.53E-02 | E4 | 7.083 | 7.78E-03 |
| TAG 50:2 | *NECTIN2* | rs8105340 | 44864520 | C | 0.074 | 0.076 | 0.056 | 1.71E-01 | 7.85E-01 | 0.096 | 0.003 | 0.023 | 8.84E-01 | 7.97E-01 | 0.155 | 0.143 | 0.035 | **5.49E-05** | **5.11E-03** | E4 | 1.005 | 3.16E-01 | E4 | 10.791 | 1.02E-03 |
| TAG 52:1 | *NECTIN2* | rs8105340 | 44864520 | C | 0.074 | 0.159 | 0.087 | 6.59E-02 | 6.50E-01 | 0.096 | 0.021 | 0.037 | 5.71E-01 | 8.10E-01 | 0.155 | 0.220 | 0.057 | **1.03E-04** | **8.88E-03** | E4 | 0.344 | 5.58E-01 | E4 | 8.596 | 3.37E-03 |
| ADMA | *NECTIN2* | rs12610605 | 44867581 | A | 0.164 | 0.100 | 0.048 | 3.69E-02 | 3.53E-01 | 0.196 | -0.003 | 0.014 | 8.62E-01 | 9.92E-01 | 0.138 | 0.101 | 0.032 | **1.42E-03** | **2.74E-02** | E4 | 0.000 | 9.84E-01 | E4 | 8.851 | 2.93E-03 |
| Aconitate | *NECTIN2* | rs519113 | 44873027 | G | 0.345 | 0.018 | 0.028 | 5.19E-01 | 8.45E-01 | 0.186 | 0.006 | 0.016 | 7.02E-01 | 9.93E-01 | 0.244 | 0.098 | 0.026 | **1.48E-04** | **1.37E-02** | E4 | 4.443 | 3.50E-02 | E4 | 9.300 | 2.29E-03 |
| Isocitrate | *NECTIN2* | rs519113 | 44873027 | G | 0.345 | 0.026 | 0.029 | 3.77E-01 | 9.92E-01 | 0.186 | 0.008 | 0.016 | 5.97E-01 | 9.95E-01 | 0.244 | 0.111 | 0.025 | **1.30E-05** | **1.21E-03** | E4 | 4.914 | 2.66E-02 | E4 | 11.833 | **5.82E-04** |
| Isocitrate | *NECTIN2* | rs2075642 | 44874210 | A | 0.135 | -0.037 | 0.039 | 3.47E-01 | 9.92E-01 | 0.233 | -0.011 | 0.014 | 4.34E-01 | 9.95E-01 | 0.231 | -0.077 | 0.024 | **1.17E-03** | **3.68E-02** | E4 | 0.790 | 3.74E-01 | E4 | 5.823 | 1.58E-02 |
| Isocitrate | *NECTIN2* | rs387976 | 44875803 | C | 0.458 | 0.030 | 0.028 | 2.77E-01 | 9.92E-01 | 0.319 | -0.006 | 0.013 | 6.54E-01 | 9.95E-01 | 0.283 | 0.078 | 0.024 | **1.19E-03** | **3.68E-02** | E4 | 1.756 | 1.85E-01 | E4 | 9.475 | 2.08E-03 |
| ADMA | *TOMM40* | rs8106922 | 44898409 | G | 0.280 | 0.010 | 0.041 | 8.11E-01 | 9.90E-01 | 0.488 | -0.012 | 0.012 | 3.18E-01 | 6.79E-01 | 0.254 | -0.092 | 0.029 | **1.40E-03** | **2.74E-02** | E4 | 4.162 | 4.13E-02 | E4 | 6.605 | 1.02E-02 |
| ADMA | *APOE* | rs440446 | 44905910 | C | 0.249 | 0.017 | 0.042 | 6.89E-01 | 9.90E-01 | 0.448 | 0.022 | 0.012 | 7.09E-02 | 6.79E-01 | 0.230 | 0.093 | 0.031 | **2.27E-03** | **3.51E-02** | E4 | 2.206 | 1.37E-01 | E4 | 4.786 | 2.87E-02 |
| ADMA | *APOE* | rs769450 | 44907187 | A | 0.288 | 0.002 | 0.041 | 9.53E-01 | 9.90E-01 | 0.492 | -0.011 | 0.012 | 3.47E-01 | 6.79E-01 | 0.242 | -0.102 | 0.029 | **5.07E-04** | **2.09E-02** | E4 | 4.305 | 3.80E-02 | E4 | 8.206 | 4.18E-03 |
| Propionate | *APOC1* | rs1064725 | 44919304 | G | 0.025 | 0.321 | 0.251 | 2.00E-01 | 8.05E-01 | 0.050 | -0.012 | 0.077 | 8.77E-01 | 1.00E+00 | 0.029 | -0.711 | 0.189 | **1.70E-04** | **1.58E-02** | E4 | 10.803 | 1.01E-03 | E4 | 11.716 | **6.20E-04** |
| Thyroxine | *APOC2, APOC4-APOC2* | rs12709889 | 44949982 | A | 0.282 | -0.015 | 0.029 | 6.13E-01 | 8.96E-01 | 0.245 | -0.004 | 0.013 | 7.48E-01 | 7.40E-01 | 0.245 | 0.076 | 0.021 | **3.36E-04** | **3.13E-02** | E4 | 6.245 | 1.25E-02 | E4 | 10.226 | 1.38E-03 |
| Uridine | *RELB* | rs12609547 | 45028751 | T | 0.390 | 0.022 | 0.027 | 4.11E-01 | 9.69E-01 | 0.407 | 0.001 | 0.013 | 9.38E-01 | 8.87E-01 | 0.459 | 0.073 | 0.020 | **2.74E-04** | **1.99E-02** | E4 | 2.341 | 1.26E-01 | E4 | 9.226 | 2.39E-03 |
| SM 22:0 | *CKM* | rs11559024 | 45317925 | C | 0.016 | 0.020 | 0.079 | 8.00E-01 | 7.24E-01 | 0.016 | -0.015 | 0.034 | 6.66E-01 | 9.82E-01 | 0.027 | -0.198 | 0.047 | **3.04E-05** | **1.58E-03** | E4 | 5.566 | 1.83E-02 | E4 | 9.800 | 1.75E-03 |
| TAG 58:11 | *CKM* | rs11559024 | 45317925 | C | 0.016 | 0.024 | 0.220 | 9.13E-01 | 9.62E-01 | 0.016 | 0.099 | 0.090 | 2.73E-01 | 3.86E-01 | 0.027 | 0.465 | 0.127 | **2.51E-04** | **2.34E-02** | E4 | 3.014 | 8.25E-02 | E4 | 5.503 | 1.90E-02 |
| TAG 56:6 | *DMPK* | rs527221 | 45772718 | C | 0.119 | 0.017 | 0.051 | 7.45E-01 | 4.39E-01 | 0.097 | 0.005 | 0.025 | 8.47E-01 | 9.79E-01 | 0.110 | 0.153 | 0.042 | **2.92E-04** | **2.72E-02** | E4 | 4.182 | 4.09E-02 | E4 | 9.182 | 2.44E-03 |
| TAG 56:6 | *DMWD* | rs8100208 | 45787357 | C | 0.122 | -0.004 | 0.051 | 9.43E-01 | 4.57E-01 | 0.103 | -0.018 | 0.024 | 4.56E-01 | 9.62E-01 | 0.124 | -0.131 | 0.039 | **8.41E-04** | **3.91E-02** | E4 | 3.900 | 4.83E-02 | E4 | 6.007 | 1.43E-02 |

All SNPs are on chromosome 19q13.32.

Abbreviations: E2 = ε2ε2 and ε2ε3 subjects; E3 = ε3ε3 subjects; E4 = ε3ε4 and ε4ε4 subjects; SNP = single-nucleotide polymorphism; POS = SNP position based on Human Genome version 38 (hg38); EA = Effect allele; EAF = Effect allele frequency; beta and se = effect size and its standard error; Magn = the group in which the magnitude of SNP effect size (i.e., absolute value of beta) is larger; $\chi^2$ = chi-square statistic corresponding to the comparison of effect sizes; TAG = triacylglycerol; PC = phosphatidylcholine; CE = cholesterol ester; SM = sphingomyelin; ADMA = asymmetric dimethylarginine.

**Table S4.** Matrix of linkage disequilibrium measures (lower-left triangle: $r^2$ and upper-right triangle: D') among SNPs whose associations identified in the E2 group.

| $r^2$\D' | rs2965101 | rs17728272 | rs2965174 | rs10402271 | rs7412 | rs5157 | rs2288912 | rs10421404 | rs7257468 | rs10413089 | rs3760627 | rs2239375 | rs123187 | rs16979759 |
|---|---|---|---|---|---|---|---|---|---|---|---|---|---|---|
| **rs2965101** | 1 | 1 | 0.434 | 0.464 | 0.204 | 0.008 | 0.017 | 0.015 | 0.024 | 0.015 | 0.024 | 0.024 | 0.129 | 1 |
| **rs17728272** | **0.652** | 1 | 1 | 0.551 | 0.676 | 0.084 | 0.074 | 0.046 | 0.032 | 0.046 | 0.032 | 0.032 | 0.229 | 1 |
| **rs2965174** | 0.064 | **0.22** | 1 | 0.002 | 0.606 | 0.003 | 0.018 | 0.017 | 0.001 | 0.017 | 0.001 | 0.001 | 0.062 | 1 |
| **rs10402271** | 0.063 | 0.058 | 0 | 1 | 0.797 | 0.024 | 0.014 | 0.52 | 0.059 | 0.52 | 0.059 | 0.059 | 0.002 | 1 |
| **rs7412** | 0.006 | 0.01 | 0.037 | 0.027 | 1 | 0.138 | 0.146 | 0.169 | 0.177 | 0.169 | 0.177 | 0.177 | 0.4 | 1 |
| **rs5157** | 0 | 0.002 | 0 | 0 | 0.002 | 1 | 0.979 | 0.943 | 0.957 | 0.943 | 0.957 | 0.957 | 0.151 | 1 |
| **rs2288912** | 0 | 0.001 | 0 | 0 | 0.002 | **0.94** | 1 | 0.944 | 0.979 | 0.944 | 0.979 | 0.979 | 0.142 | 1 |
| **rs10421404** | 0 | 0.001 | 0 | 0.033 | 0.01 | **0.205** | **0.21** | 1 | 1 | 1 | 1 | 1 | 0.408 | 1 |
| **rs7257468** | 0 | 0 | 0 | 0.002 | 0.003 | **0.827** | **0.883** | **0.255** | 1 | 1 | 1 | 1 | 0.167 | 1 |
| **rs10413089** | 0 | 0.001 | 0 | 0.033 | 0.01 | **0.205** | **0.21** | **1** | **0.255** | 1 | 1 | 1 | 0.408 | 1 |
| **rs3760627** | 0 | 0 | 0 | 0.002 | 0.003 | **0.827** | **0.883** | **0.255** | **1** | **0.255** | 1 | 1 | 0.167 | 1 |
| **rs2239375** | 0 | 0 | 0 | 0.002 | 0.003 | **0.827** | **0.883** | **0.255** | **1** | **0.255** | **1** | 1 | 0.167 | 1 |
| **rs123187** | 0.004 | 0.009 | 0.003 | 0 | 0.02 | 0.011 | 0.01 | 0.019 | 0.012 | 0.019 | 0.012 | 0.012 | 1 | 1 |
| **rs16979759** | 0.002 | 0.002 | 0.004 | 0.003 | 0 | 0.004 | 0.004 | 0.001 | 0.004 | 0.001 | 0.004 | 0.004 | 0.003 | 1 |

SNP-pairs with $r^2 \geq 0.1$ (bold font in the lower-left triangle) are in linkage disequilibrium ($P<0.0001$) in the CEU population (i.e., Utah Residents with Northern and Western European Ancestry).

**Table S5.** Matrix of linkage disequilibrium measures (lower-left tringle: $r^2$ and upper-right triangle: D') among SNPs whose associations identified in the E3 group.

| $r^2$\D' | rs5157 | rs12721109 | rs5167 | rs2288912 | rs7257468 | rs12709889 | rs3760627 | rs2239375 | rs4884 | rs11083777 | rs16939 |
|---|---|---|---|---|---|---|---|---|---|---|---|
| **rs5157** | 1 | 1 | 0.972 | 0.979 | 0.957 | 0.965 | 0.957 | 0.957 | 0.05 | 0.283 | 0.125 |
| **rs12721109** | 0.018 | 1 | 1 | 1 | 1 | 1 | 1 | 1 | 1 | 1 | 0.383 |
| **rs5167** | **0.557** | 0.008 | 1 | 0.972 | 0.867 | 1 | 0.867 | 0.867 | 0.148 | 0.015 | 0.156 |
| **rs2288912** | **0.94** | 0.018 | **0.569** | 1 | 0.979 | 1 | 0.979 | 0.979 | 0.028 | 0.378 | 0.139 |
| **rs7257468** | **0.827** | 0.02 | **0.49** | **0.883** | 1 | 1 | 1 | 1 | 0.033 | 0.458 | 0.15 |
| **rs12709889** | **0.403** | 0.006 | **0.733** | **0.441** | **0.478** | 1 | 1 | 1 | 0.379 | 0.302 | 0.194 |
| **rs3760627** | **0.827** | 0.02 | **0.49** | **0.883** | **1** | **0.478** | 1 | 1 | 0.033 | 0.458 | 0.15 |
| **rs2239375** | **0.827** | 0.02 | **0.49** | **0.883** | **1** | **0.478** | **1** | 1 | 0.033 | 0.458 | 0.15 |
| **rs4884** | 0.001 | 0.036 | 0.005 | 0 | 0.001 | 0.023 | 0.001 | 0.001 | 1 | 0.201 | 0.041 |
| **rs11083777** | 0.008 | 0.002 | 0 | 0.014 | 0.02 | 0.004 | 0.02 | 0.02 | 0.002 | 1 | 0.068 |
| **rs16939** | 0.012 | 0.003 | 0.011 | 0.014 | 0.015 | 0.012 | 0.015 | 0.015 | 0.001 | 0 | 1 |

SNP-pairs with $r^2 \geq 0.1$ (bold font in the lower-left triangle) are in linkage disequilibrium ($P<0.0001$) in the CEU population (i.e., Utah Residents with Northern and Western European Ancestry).

**Table S6.** Matrix of linkage disequilibrium measures (lower-left tringle: $r^2$ and upper-right triangle: D') among SNPs whose associations identified in the E4 group.

| $r^2$\D' | rs1871046 | rs8105340 | rs12610605 | rs519113 | rs2075642 | rs387976 | rs8106922 | rs440446 | rs769450 | rs1064725 | rs12709889 | rs12609547 | rs11559024 | rs527221 | rs8100208 |
|---|---|---|---|---|---|---|---|---|---|---|---|---|---|---|---|
| **rs1871046** | 1 | 0.461 | 0.8 | 0.278 | 0.933 | 0.265 | 0.777 | 0.529 | 0.777 | 0.175 | 0.11 | 0.003 | 0.434 | 0.116 | 0.214 |
| **rs8105340** | 0.014 | 1 | 1 | 1 | 1 | 1 | 0.073 | 0.738 | 0.073 | 1 | 0.083 | 0.088 | 0.105 | 0.411 | 0.476 |
| **rs12610605** | **0.217** | 0.022 | 1 | 1 | 1 | 1 | 0.832 | 0.747 | 0.832 | 0.574 | 0.054 | 0.107 | 1 | 0.202 | 0.29 |
| **rs519113** | 0.013 | **0.381** | 0.058 | 1 | 1 | 0.416 | 0.501 | 0.064 | 0.446 | 0.213 | 0.005 | 0.094 | 0.157 | 0.099 | 0.126 |
| **rs2075642** | **0.128** | 0.032 | 0.05 | 0.084 | 1 | 1 | 0.73 | 0.673 | 0.73 | 0.686 | 0.149 | 0.192 | 1 | 0.411 | 0.476 |
| **rs387976** | 0.057 | **0.266** | **0.417** | **0.121** | **0.12** | 1 | 0.53 | 0.176 | 0.53 | 0.784 | 0.01 | 0.058 | 0.351 | 0.277 | 0.197 |
| **rs8106922** | **0.206** | 0.001 | 0.08 | 0.049 | **0.23** | 0.078 | 1 | 0.964 | 0.979 | 1 | 0.128 | 0.057 | 0.479 | 0.023 | 0.132 |
| **rs440446** | **0.267** | 0.037 | **0.181** | 0.001 | 0.07 | 0.024 | **0.331** | 1 | 1 | 1 | 0.185 | 0.054 | 0.371 | 0.018 | 0.04 |
| **rs769450** | **0.206** | 0.001 | 0.08 | 0.039 | **0.23** | 0.078 | **0.958** | **0.356** | 1 | 1 | 0.128 | 0.057 | 0.479 | 0.023 | 0.132 |
| **rs1064725** | 0.005 | 0.01 | 0.005 | 0.012 | 0.01 | 0.022 | 0.051 | **0.143** | 0.051 | 1 | 0.511 | 0.29 | 0.351 | 0.057 | 0.047 |
| **rs12709889** | 0.002 | 0.002 | 0 | 0 | 0.016 | 0 | 0.01 | 0.007 | 0.01 | 0.008 | 1 | 0.022 | 1 | 0.083 | 0.083 |
| **rs12609547** | 0 | 0.001 | 0.002 | 0.002 | 0.011 | 0.001 | 0.002 | 0.001 | 0.002 | 0.006 | 0 | 1 | 0.623 | 0.175 | 0.267 |
| **rs11559024** | 0.003 | 0.002 | 0.005 | 0 | 0.007 | 0.001 | 0.004 | 0.006 | 0.004 | 0.039 | 0.01 | 0.011 | 1 | 1 | 1 |
| **rs527221** | 0.001 | 0.002 | 0.001 | 0.003 | 0.004 | 0.015 | 0 | 0 | 0 | 0.003 | 0 | 0.003 | 0.002 | 1 | 1 |
| **rs8100208** | 0.003 | 0.003 | 0.002 | 0.005 | 0.006 | 0.009 | 0.001 | 0 | 0.001 | 0.002 | 0.002 | 0.008 | 0.003 | **0.879** | 1 |

SNP-pairs with $r^2 \geq 0.1$ (bold font in the lower-left triangle) are in linkage disequilibrium ($P<0.0001$) in the CEU population (i.e., Utah Residents with Northern and Western European Ancestry).

**Table S7.** Interaction analysis of group-specific associations primarily identified in the E2 vs. E3, E2 vs. E4, or E4 vs. E3 comparisons.

| Phenotype | Gene | SNP | POS | EA | EAF | SNP | | | SNP:*APOE* status | | |
|---|---|---|---|---|---|---|---|---|---|---|---|
| | | | | | | beta | se | p-value | beta | se | p-value |
| **Group-specific associations identified in E2 vs. E3** | | | | | | | | | | | |
| Glutamic acid | *APOC2,APOC4-APOC2* | rs10421404 | 44949588 | A | 0.173 | 0.036 | 0.042 | **3.93E-01** | -0.331 | 0.097 | **6.28E-04** |
| Glutamic acid | *CLPTM1* | rs10413089 | 44952331 | C | 0.174 | 0.037 | 0.042 | **3.82E-01** | -0.332 | 0.097 | **6.08E-04** |
| Dimethylglycine | *CKM* | rs16979759 | 45327876 | G | 0.018 | -0.122 | 0.064 | **5.63E-02** | 1.041 | 0.166 | **3.54E-10** |
| **Group-specific associations identified in E2 vs. E4** | | | | | | | | | | | |
| TAG 56:5 | *BCL3* | rs2965101 | 44734556 | C | 0.345 | 0.130 | 0.030 | **1.50E-05** | -0.198 | 0.041 | **1.14E-06** |
| TAG 56:5 | *BCL3* | rs17728272 | 44737114 | T | 0.240 | 0.111 | 0.030 | **1.67E-04** | -0.166 | 0.042 | **6.30E-05** |
| LPE 16:0 | *APOC2,APOC4-APOC2* | rs7257468 | 44949887 | T | 0.485 | -0.137 | 0.041 | **9.53E-04** | 0.193 | 0.051 | **1.49E-04** |
| LPE 18:0 | *APOC2,APOC4-APOC2* | rs7257468 | 44949887 | T | 0.485 | -0.139 | 0.045 | **1.86E-03** | 0.211 | 0.055 | **1.19E-04** |
| LPE 20:4 | *APOC2,APOC4-APOC2* | rs7257468 | 44949887 | T | 0.485 | -0.125 | 0.034 | **2.96E-04** | 0.180 | 0.042 | **2.10E-05** |
| LPE 16:0 | *CLPTM1* | rs3760627 | 44953923 | C | 0.485 | -0.131 | 0.041 | **1.50E-03** | 0.186 | 0.050 | **2.24E-04** |
| LPE 18:0 | *CLPTM1* | rs3760627 | 44953923 | C | 0.485 | -0.132 | 0.044 | **3.00E-03** | 0.203 | 0.054 | **1.88E-04** |
| LPE 20:4 | *CLPTM1* | rs3760627 | 44953923 | C | 0.485 | -0.122 | 0.034 | **3.65E-04** | 0.175 | 0.042 | **3.00E-05** |
| LPE 16:0 | *CLPTM1* | rs2239375 | 44956594 | C | 0.490 | -0.131 | 0.041 | **1.53E-03** | 0.178 | 0.050 | **4.27E-04** |
| LPE 18:0 | *CLPTM1* | rs2239375 | 44956594 | C | 0.490 | -0.132 | 0.044 | **3.04E-03** | 0.200 | 0.054 | **2.41E-04** |
| LPE 20:4 | *CLPTM1* | rs2239375 | 44956594 | C | 0.490 | -0.122 | 0.034 | **3.77E-04** | 0.168 | 0.042 | **6.39E-05** |
| PC 38:6 | *CKM* | rs123187 | 45327689 | A | 0.380 | 0.098 | 0.031 | **1.60E-03** | -0.123 | 0.037 | **8.49E-04** |
| **Group-specific associations identified in E4 vs. E3** | | | | | | | | | | | |
| PC 32:1 | *NECTIN2* | rs8105340 | 44864520 | C | 0.110 | -0.036 | 0.034 | **2.83E-01** | 0.248 | 0.063 | **8.17E-05** |
| PC 32:0 | *NECTIN2* | rs8105340 | 44864520 | C | 0.110 | -0.008 | 0.017 | **6.24E-01** | 0.126 | 0.032 | **8.35E-05** |
| PC 34:1 | *NECTIN2* | rs8105340 | 44864520 | C | 0.110 | -0.017 | 0.011 | **1.12E-01** | 0.095 | 0.020 | **3.06E-06** |
| Isocitrate | *NECTIN2* | rs519113 | 44873027 | G | 0.200 | 0.009 | 0.016 | **5.53E-01** | 0.099 | 0.031 | **1.13E-03** |
| Propionate | *APOC1* | rs1064725 | 44919304 | G | 0.045 | -0.015 | 0.078 | **8.45E-01** | -0.753 | 0.197 | **1.28E-04** |

All SNPs are on chromosome 19q13.32.

Abbreviations: E2 = ε2ε2 and ε2ε3 subjects; E3 = ε3ε3 subjects; E4 = ε3ε4 and ε4ε4 subjects; *APOE* = apolipoprotein E; SNP = single-nucleotide polymorphism; POS = SNP position based on Human Genome version 38 (hg38); EA = Effect allele; EAF = Effect allele frequency; beta and se = effect size and its standard error; LPE = lysophosphatidylethanolamine; PC = phosphatidylcholine; TAG = triacylglycerol.

**Table S8.** Mean, standard error, 95% confidence interval, minimum, and maximum values of metabolites with group-specific associations.

| Phenotype | E2 | | | | | | E3 | | | | | | E4 | | | | | |
|---|---|---|---|---|---|---|---|---|---|---|---|---|---|---|---|---|---|---|
| | mean | se | 95%CI-L | 95%CI-U | min | max | mean | se | 95%CI-L | 95%CI-U | min | max | mean | se | 95%CI-L | 95%CI-U | min | max |
| **E2-specific** | | | | | | | | | | | | | | | | | | |
| TAG 56:5 | 0.955 | 0.021 | 0.913 | 0.996 | 0.297 | 1.866 | 0.886 | 0.009 | 0.870 | 0.903 | 0.326 | 2.245 | 0.928 | 0.016 | 0.897 | 0.959 | 0.328 | 1.867 |
| Glutamic acid | 8.860 | 0.330 | 8.214 | 9.506 | 1.107 | 27.443 | 8.503 | 0.143 | 8.223 | 8.783 | 1.106 | 31.682 | 8.724 | 0.254 | 8.225 | 9.222 | 1.712 | 29.158 |
| LPE 16:0 | 1.449 | 0.037 | 1.378 | 1.521 | 0.369 | 4.501 | 1.396 | 0.019 | 1.359 | 1.434 | 0.364 | 5.413 | 1.411 | 0.031 | 1.351 | 1.472 | 0.477 | 4.688 |
| LPE 18:0 | 1.831 | 0.057 | 1.718 | 1.943 | 0.535 | 6.430 | 1.722 | 0.022 | 1.679 | 1.764 | 0.489 | 7.352 | 1.840 | 0.045 | 1.752 | 1.928 | 0.579 | 5.933 |
| LPE 20:4 | 1.154 | 0.025 | 1.104 | 1.203 | 0.342 | 2.481 | 1.131 | 0.012 | 1.108 | 1.155 | 0.373 | 2.956 | 1.164 | 0.021 | 1.123 | 1.205 | 0.529 | 3.376 |
| PC 38:6 | 0.873 | 0.014 | 0.845 | 0.901 | 0.471 | 1.417 | 0.847 | 0.006 | 0.835 | 0.859 | 0.411 | 1.446 | 0.873 | 0.012 | 0.850 | 0.897 | 0.419 | 1.596 |
| Dimethylglycine | 1.383 | 0.248 | 0.897 | 1.868 | 0.456 | 58.747 | 1.132 | 0.014 | 1.105 | 1.159 | 0.324 | 5.286 | 1.097 | 0.024 | 1.049 | 1.144 | 0.452 | 5.577 |
| **E4-specific** | | | | | | | | | | | | | | | | | | |
| PC 32:1 | 1.027 | 0.033 | 0.961 | 1.093 | 0.294 | 2.999 | 1.005 | 0.016 | 0.973 | 1.036 | 0.241 | 4.345 | 1.044 | 0.029 | 0.988 | 1.100 | 0.302 | 3.940 |
| PC 32:0 | 1.021 | 0.014 | 0.994 | 1.048 | 0.564 | 1.514 | 1.023 | 0.007 | 1.009 | 1.037 | 0.418 | 1.985 | 1.009 | 0.013 | 0.984 | 1.035 | 0.471 | 2.015 |
| PC 34:1 | 1.047 | 0.010 | 1.027 | 1.067 | 0.662 | 1.440 | 1.040 | 0.005 | 1.031 | 1.049 | 0.561 | 1.591 | 1.053 | 0.008 | 1.036 | 1.069 | 0.675 | 1.504 |
| Isocitrate | 1.168 | 0.024 | 1.121 | 1.215 | 0.581 | 3.203 | 1.173 | 0.011 | 1.151 | 1.194 | 0.367 | 4.035 | 1.188 | 0.020 | 1.148 | 1.227 | 0.565 | 3.999 |
| Propionate | 1.188 | 0.045 | 1.100 | 1.275 | 0.021 | 3.566 | 1.155 | 0.020 | 1.116 | 1.195 | 0.020 | 4.769 | 1.133 | 0.033 | 1.068 | 1.198 | 0.031 | 3.771 |

Abbreviations: E2 = ε2ε2 and ε2ε3 subjects; E3 = ε3ε3 subjects; E4 = ε3ε4 and ε4ε4 subjects; se = standard error; 95%CI-L and 95%CI-U = 95% confidence interval lower and upper bounds; min = minimum; max = maximum.

**Table S9.** Significant associations identified in the pooled sample of the E2, E3, and E4 groups.

| Phenotype | Gene | SNP | POS | EA | EAF | beta | se | p-value | q-value |
|---|---|---|---|---|---|---|---|---|---|
| TAG 56:4 | *APOE* | rs7412 | 44908822 | T | 0.066 | 0.085 | 0.023 | **2.76E-04** | **1.30E-02** |
| TAG 56:3 | *APOE* | rs7412 | 44908822 | T | 0.066 | 0.123 | 0.029 | **2.21E-05** | **4.42E-04** |
| CE 20:3 | *APOC4,APOC4-APOC2* | rs5157 | 44943904 | T | 0.518 | -0.047 | 0.014 | **5.87E-04** | **9.47E-03** |
| TAG 56:4 | *APOC4,APOC4-APOC2* | rs12721109 | 44943964 | A | 0.021 | 0.202 | 0.041 | **9.13E-07** | **8.58E-05** |
| CE 20:3 | *APOC4,APOC4-APOC2* | rs5167 | 44945208 | G | 0.344 | 0.055 | 0.015 | **1.51E-04** | **6.80E-03** |
| CE 20:3 | *APOC2,APOC4,APOC4-APOC2* | rs2288912 | 44945942 | G | 0.477 | 0.046 | 0.014 | **8.43E-04** | **9.47E-03** |
| CE 20:3 | *APOC2,APOC4-APOC2* | rs7257468 | 44949887 | T | 0.443 | 0.041 | 0.014 | **3.58E-03** | **2.17E-02** |
| CE 20:3 | *CLPTM1* | rs3760627 | 44953923 | C | 0.443 | 0.040 | 0.014 | **3.79E-03** | **2.17E-02** |
| CE 20:3 | *CLPTM1* | rs2239375 | 44956594 | C | 0.445 | 0.040 | 0.014 | **3.86E-03** | **2.17E-02** |

All SNPs are on chromosome 19q13.32.

Abbreviations: E2 = ε2ε2 and ε2ε3 subjects; E3 = ε3ε3 subjects; E4 = ε3ε4 and ε4ε4 subjects; SNP = single-nucleotide polymorphism; POS = SNP position based on Human Genome version 38 (hg38); EA = Effect allele; EAF = Effect allele frequency; beta and se = effect size and its standard error; CE = cholesterol ester; TAG = triacylglycerol.

**Table S10.** Associations of rs429358 and rs7412 (i.e., *APOE* SNPs) with 32 metabolites of interest in the pooled sample of the E2, E3, and E4 groups.

| Phenotype | rs429358 (EA=C, EAF=0.11) | | | | rs7412 (EA=T, EAF=0.06) | | | |
|---|---|---|---|---|---|---|---|---|
| | beta | se | p-value | q-value | beta | se | p-value | q-value |
| Aconitate | 0.017 | 0.015 | 2.41E-01 | 6.09E-01 | -0.008 | 0.019 | 6.91E-01 | 6.09E-01 |
| Isocitrate | 0.017 | 0.015 | 2.72E-01 | 9.74E-01 | -0.002 | 0.019 | 9.26E-01 | 9.74E-01 |
| Sucrose | -0.068 | 0.053 | 1.99E-01 | 7.78E-01 | 0.035 | 0.069 | 6.09E-01 | 9.81E-01 |
| Uridine | 0.005 | 0.015 | 7.69E-01 | 6.62E-01 | 0.013 | 0.020 | 5.10E-01 | 6.53E-01 |
| Propionate | -0.012 | 0.043 | 7.82E-01 | 9.90E-01 | 0.038 | 0.056 | 4.95E-01 | 9.89E-01 |
| Asparagine | -0.025 | 0.024 | 2.83E-01 | 9.26E-01 | 0.014 | 0.030 | 6.51E-01 | 9.96E-01 |
| Aspartate | 0.018 | 0.036 | 6.27E-01 | 7.23E-01 | 0.037 | 0.048 | 4.38E-01 | 7.23E-01 |
| Glutamic acid | -0.011 | 0.035 | 7.50E-01 | 9.15E-01 | 0.053 | 0.045 | 2.43E-01 | 7.38E-01 |
| Xanthosine | -0.024 | 0.017 | 1.56E-01 | 9.07E-01 | -0.004 | 0.022 | 8.50E-01 | 9.82E-01 |
| Dimethylglycine | -0.044 | 0.020 | 2.42E-02 | 2.06E-01 | 0.059 | 0.025 | 1.95E-02 | 2.06E-01 |
| Thyroxine | 0.016 | 0.013 | 2.45E-01 | 3.79E-01 | 0.011 | 0.017 | 5.17E-01 | 4.13E-01 |
| ADMA | 0.010 | 0.015 | 5.25E-01 | 4.81E-01 | 0.031 | 0.020 | 1.15E-01 | 2.36E-01 |
| LPE 16:0 | -0.009 | 0.023 | 7.00E-01 | 4.37E-01 | 0.039 | 0.029 | 1.79E-01 | 3.03E-01 |
| LPE 18:0 | 0.030 | 0.022 | 1.84E-01 | 9.13E-01 | 0.048 | 0.029 | 9.34E-02 | 8.29E-01 |
| LPE 20:4 | 0.029 | 0.019 | 1.25E-01 | 5.76E-01 | 0.017 | 0.024 | 4.84E-01 | 8.48E-01 |
| PC 32:1 | 0.008 | 0.025 | 7.65E-01 | 6.30E-01 | 0.011 | 0.032 | 7.27E-01 | 6.30E-01 |
| PC 32:0 | -0.024 | 0.013 | 6.37E-02 | 3.33E-01 | 0.020 | 0.016 | 2.28E-01 | 6.85E-01 |
| PC 34:1 | 0.002 | 0.008 | 8.11E-01 | 7.99E-01 | 0.003 | 0.011 | 7.40E-01 | 7.98E-01 |
| PC 38:6 | 0.017 | 0.014 | 2.21E-01 | 9.90E-01 | 0.020 | 0.018 | 2.60E-01 | 9.90E-01 |
| SM 18:0 | -0.027 | 0.014 | 5.46E-02 | 2.13E-01 | -0.016 | 0.018 | 3.52E-01 | 3.39E-01 |
| SM 22:0 | -0.027 | 0.011 | 1.23E-02 | 3.86E-01 | -0.012 | 0.014 | 3.83E-01 | 9.61E-01 |
| CE 20:3 | -0.009 | 0.021 | 6.82E-01 | 4.02E-01 | -0.067 | 0.027 | 1.43E-02 | 7.15E-02 |
| TAG 50:2 | 0.011 | 0.017 | 5.29E-01 | 9.10E-01 | -0.001 | 0.022 | 9.54E-01 | 9.35E-01 |
| TAG 52:1 | 0.035 | 0.028 | 2.08E-01 | 4.81E-01 | 0.028 | 0.036 | 4.31E-01 | 5.45E-01 |
| TAG 54:6 | 0.010 | 0.018 | 5.65E-01 | 5.80E-01 | 0.058 | 0.023 | 1.04E-02 | 2.30E-01 |
| TAG 54:5 | 0.010 | 0.018 | 5.65E-01 | 5.80E-01 | 0.058 | 0.023 | 1.04E-02 | 2.30E-01 |
| TAG 54:4 | 0.007 | 0.014 | 6.40E-01 | 9.29E-01 | 0.039 | 0.019 | 3.38E-02 | 4.78E-01 |
| TAG 56:6 | 0.006 | 0.019 | 7.55E-01 | 3.49E-01 | 0.067 | 0.024 | 5.65E-03 | 5.29E-02 |
| TAG 56:5 | -0.001 | 0.017 | 9.74E-01 | 9.84E-01 | 0.063 | 0.022 | 4.22E-03 | 1.98E-01 |
| TAG 56:4 | 0.009 | 0.018 | 6.27E-01 | 9.18E-01 | 0.085 | 0.023 | 2.76E-04 | **1.30E-02** |
| TAG 56:3 | 0.013 | 0.023 | 5.70E-01 | 1.69E-01 | 0.123 | 0.029 | 2.21E-05 | **4.42E-04** |
| TAG 58:11 | 0.047 | 0.029 | 1.06E-01 | 6.65E-01 | 0.087 | 0.037 | 1.95E-02 | 4.58E-01 |

Both rs429358 and rs7412 are on chromosome 19q13.32.

Abbreviations: E2 = ε2ε2 and ε2ε3 subjects; E3 = ε3ε3 subjects; E4 = ε3ε4 and ε4ε4 subjects; *APOE* = apolipoprotein E; SNP = single-nucleotide polymorphism; EA = Effect allele; EAF = Effect allele frequency; beta and se = effect size and its standard error; ADMA = asymmetric dimethylarginine; LPE = lysophosphatidylethanolamine; PC = phosphatidylcholine; SM = sphingomyelin; CE = cholesterol ester; TAG = triacylglycerol.

**Table S11.** *APOE*-allele-specific clustering of genes identified for all metabolites, lipid metabolites, and non-lipid metabolites.

| Group | *BCL3* | *BCAM* | *NECTIN2* | *TOMM40* | *APOE* | *APOC1* | *APOC4* | *APOC2* | *CLPTM1* | *RELB* | *CKM* | *EML2* | *DMPK* | *DMWD* |
|---|---|---|---|---|---|---|---|---|---|---|---|---|---|---|
| **All Metabolites** | | | | | | | | | | | | | | |
| **E2** | x | x | x | | x | | x | x | x | | x | | | |
| **E3** | | | | | | | x | x | x | | x | x | x | |
| **E4** | | | x | x | x | x | x | x | | x | x | | x | x |
| **Lipid Metabolites** | | | | | | | | | | | | | | |
| **E2** | x | | | | x | | x | x | x | | x | | | |
| **E3** | | | | | | | x | x | x | | | | | |
| **E4** | | | x | | | | | | | | x | | x | x |
| **Polar Metabolites** | | | | | | | | | | | | | | |
| **E2** | | x | x | | | | x | x | x | | x | | | |
| **E3** | | | | | | | | | | | x | x | x | |
| **E4** | | | x | x | x | x | x | x | | x | | | | |

Abbreviations: E2 = ε2ε2 and ε2ε3 subjects; E3 = ε3ε3 subjects; E4 = ε3ε4 and ε4ε4 subjects.
Green highlights indicate genes whose variants are uniquely associated with plasma metabolites in only one of the three analyzed groups.

## Figures

**Figure S1.** Distributions of plasma concentrations of metabolites with group-specific associations.

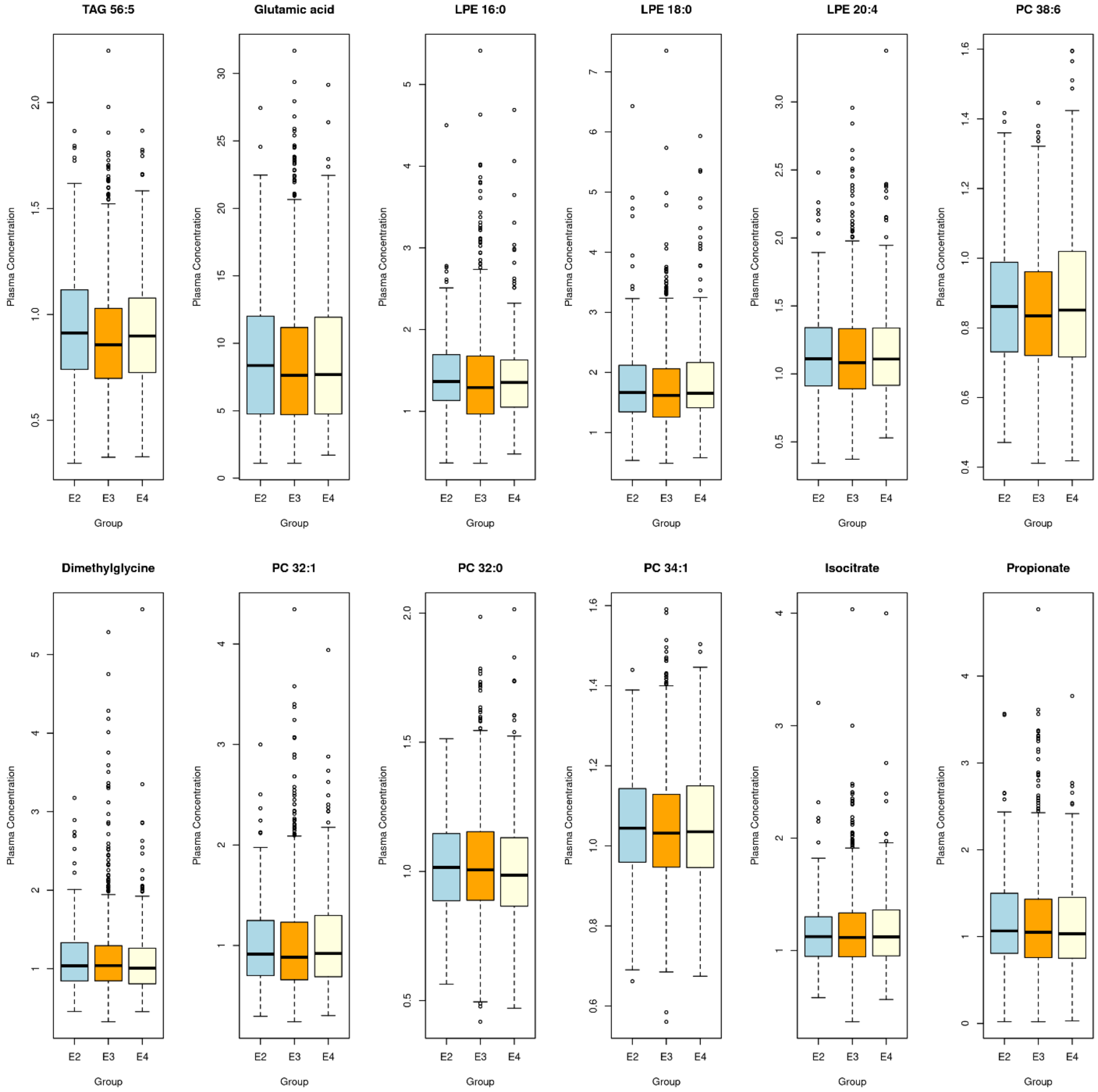


Abbreviations: E2 = ε2ε2 and ε2ε3 subjects; E3 = ε3ε3 subjects; E4 = ε3ε4 and ε4ε4 subjects; PC = phosphatidylcholine; LPE = lysophosphatidylethanolamine; TAG = triacylglycerol.

**Figure S2.** Pathway enrichment for lipid metabolites with significant genetic associations in the E2 group.

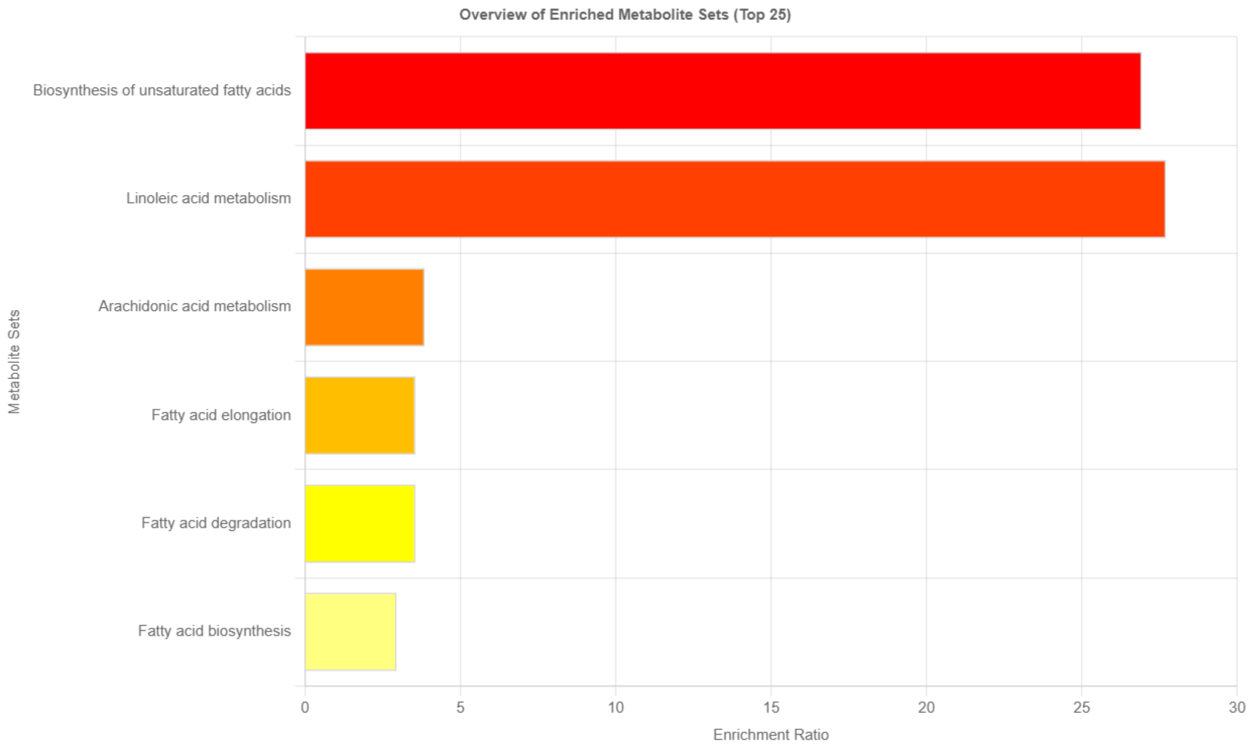

**Figure S3.** Pathway enrichment for polar metabolites with significant genetic associations in the E2 group.

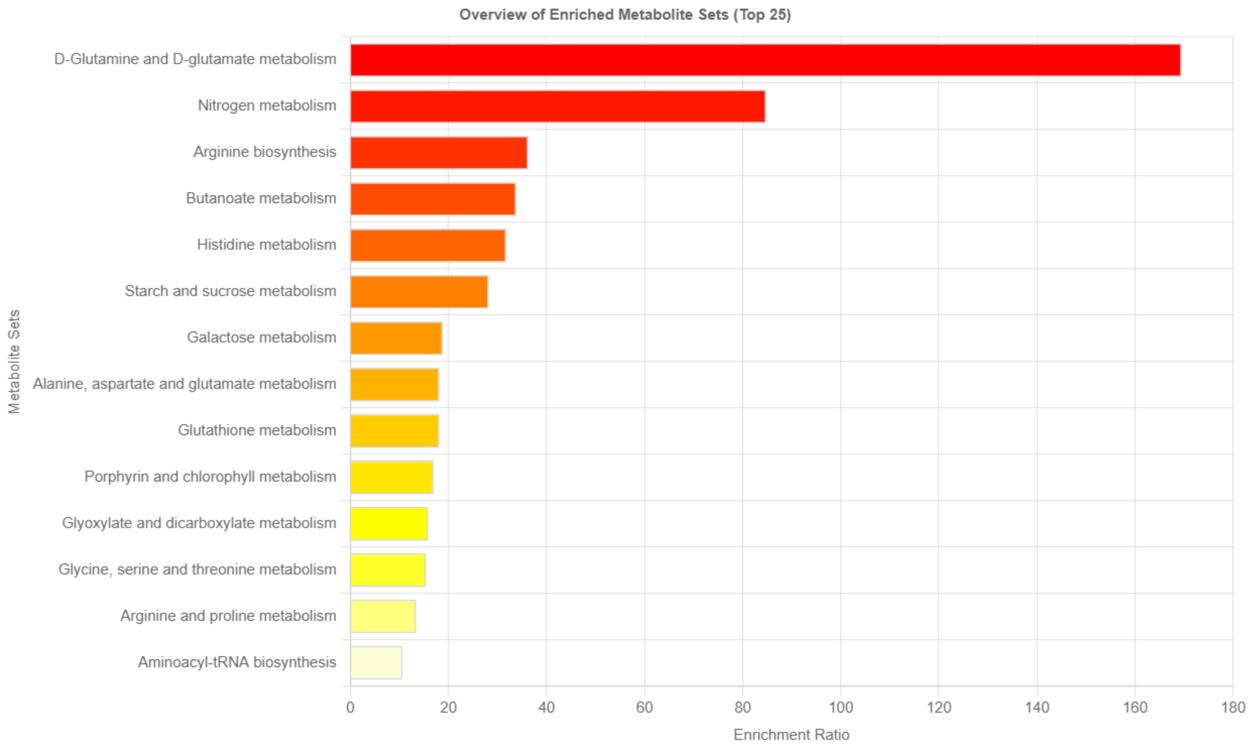

**Figure S4.** Pathway enrichment for lipid metabolites with significant genetic associations in the E3 group.

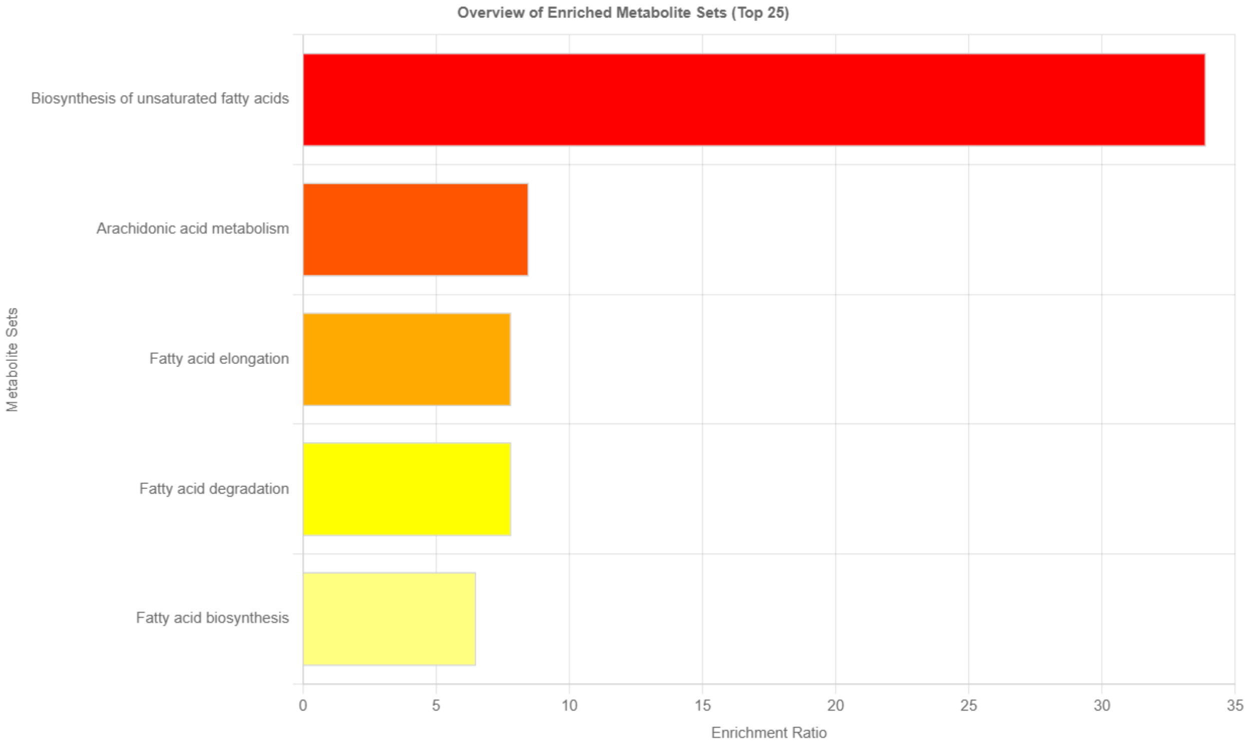

**Figure S5.** Pathway enrichment for polar metabolites with significant genetic associations in the E3 group.

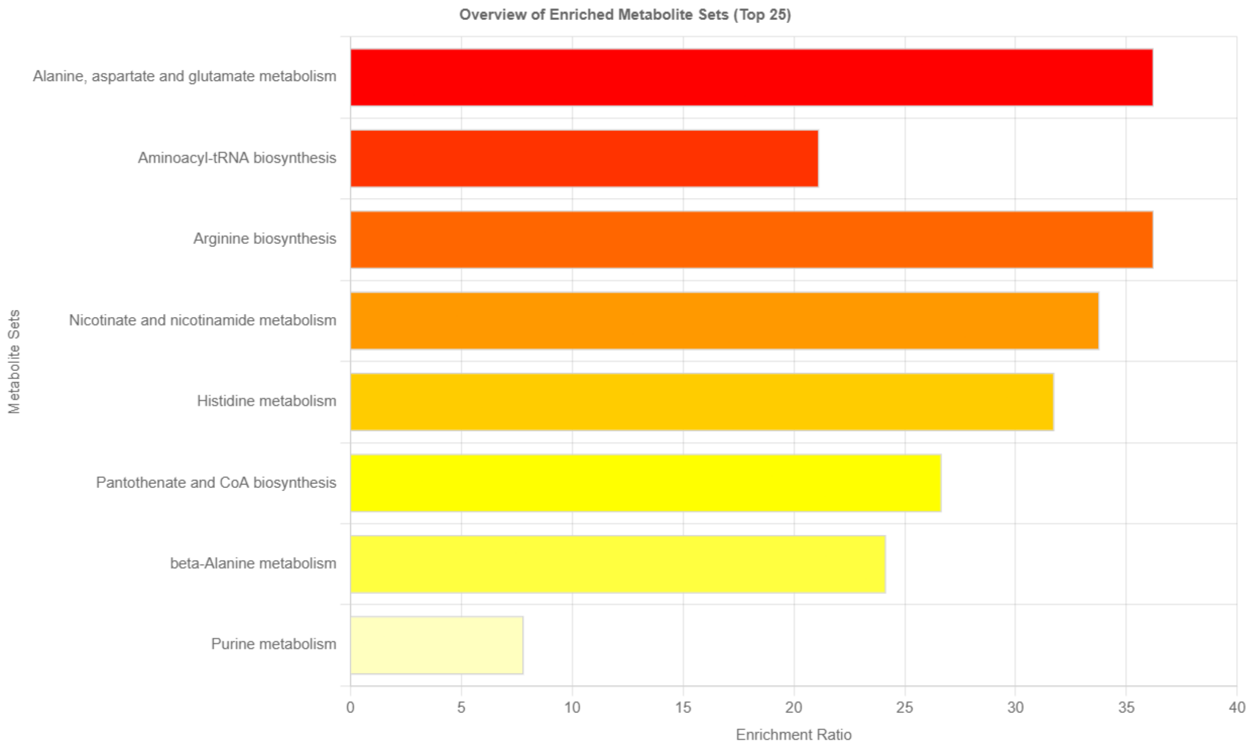

**Figure S6.** Pathway enrichment for lipid metabolites with significant genetic associations in the E4 group.

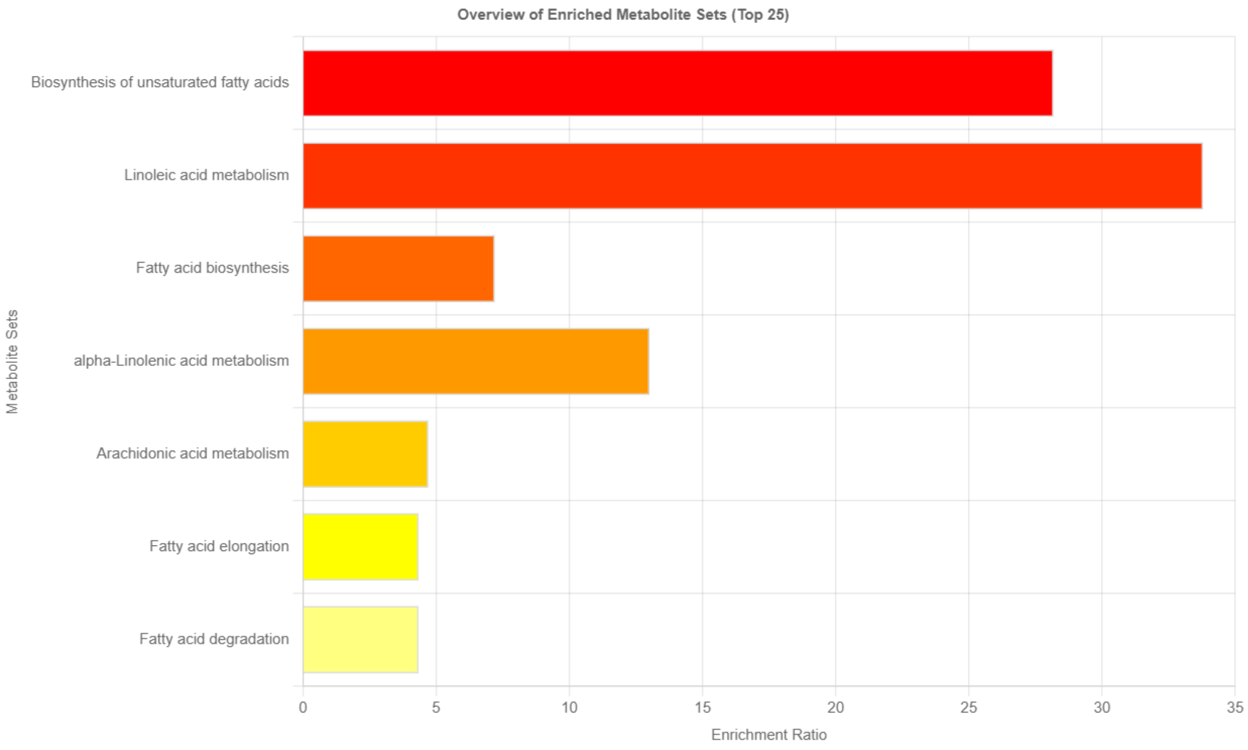

**Figure S7.** Pathway enrichment for polar metabolites with significant genetic associations in the E4 group.

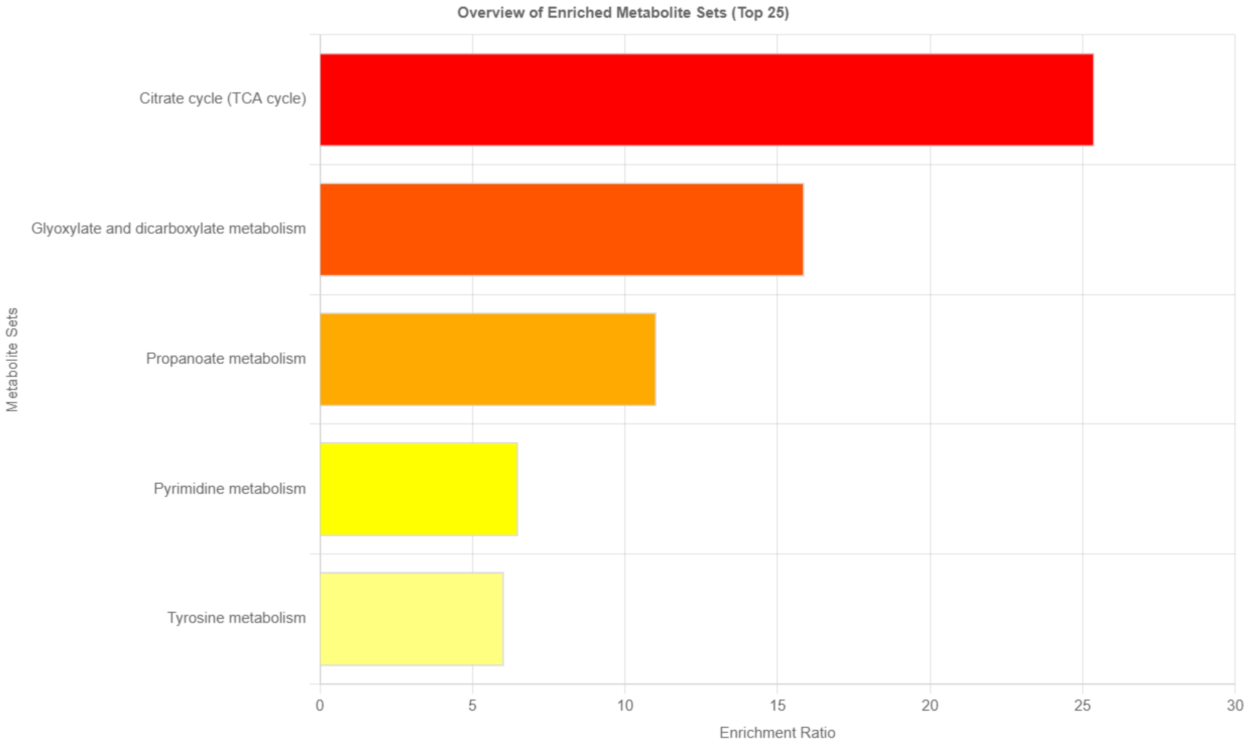

Supplement: Supplementary file 1 [file Data_Sheet_1.PDF]
